# Safety of psychotropic medications in people with COVID-19: evidence review and practical recommendations

**DOI:** 10.1186/s12916-020-01685-9

**Published:** 2020-07-15

**Authors:** Giovanni Ostuzzi, Davide Papola, Chiara Gastaldon, Georgios Schoretsanitis, Federico Bertolini, Francesco Amaddeo, Alessandro Cuomo, Robin Emsley, Andrea Fagiolini, Giuseppe Imperadore, Taishiro Kishimoto, Giulia Michencigh, Michela Nosé, Marianna Purgato, Dursun Serdar, Brendon Stubbs, David Taylor, Graham Thornicroft, Philip B. Ward, Christoph Hiemke, Christoph U. Correll, Corrado Barbui

**Affiliations:** 1 WHO Collaborating Centre for Research and Training in Mental Health and Service Evaluation, Department of Neuroscience, Biomedicine and Movement Sciences, Section of Psychiatry, University of Verona, Verona, Italy; 2 Department of Psychiatry, The Zucker Hillside Hospital, Northwell Health, Glen Oaks, NY USA; 3 Department of Molecular Medicine, University of Siena, Siena, Italy; 4 Department of Psychiatry, Faculty of Medicine and Health Sciences, Stellenbosch University, Tygerberg Campus, Cape Town, 8000 South Africa; 5 Azienda ULSS 9 Scaligera, Verona, Italy; 6 Department of Neuropsychiatry, Keio University School of Medicine, Tokyo, Japan; 7 Department of Psychiatry, University of Alberta, Edmonton, Alberta Canada; 8 Department of Psychological Medicine, Institute of Psychiatry, Psychology, and Neuroscience, King’s College London, London, UK; 9 Physiotherapy Department, South London and Maudsley National Health Services Foundation Trust, London, UK; 10 Pharmacy Department, Maudsley Hospital, London, UK; 11 Centre for Global Mental Health and Centre for Implementation Science, Institute of Psychiatry, Psychology and Neuroscience, King’s College London, London, UK; 12 School of Psychiatry, UNSW Sydney and Schizophrenia Research Unit, Ingham Institute of Applied Medical Research, Liverpool, NSW Australia; 13 Department of Psychiatry and Psychotherapy, University Medical Center of Mainz, Mainz, Germany; 14 Department of Psychiatry and Molecular Medicine, Zucker School of Medicine at Hofstra/Northwell, Hempstead, NY USA; 15 Department of Child and Adolescent Psychiatry, Charité Universitätsmedizin Berlin, Berlin, Germany

**Keywords:** Novel coronavirus, COVID-19, Psychopharmacology, Psychiatric comorbidity, Drug–drug interactions

## Abstract

**Background:**

The novel coronavirus pandemic calls for a rapid adaptation of conventional medical practices to meet the evolving needs of such vulnerable patients. People with coronavirus disease (COVID-19) may frequently require treatment with psychotropic medications, but are at the same time at higher risk for safety issues because of the complex underlying medical condition and the potential interaction with medical treatments.

**Methods:**

In order to produce evidence-based practical recommendations on the optimal management of psychotropic medications in people with COVID-19, an international, multi-disciplinary working group was established. The methodology of the WHO Rapid Advice Guidelines in the context of a public health emergency and the principles of the AGREE statement were followed. Available evidence informing on the risk of respiratory, cardiovascular, infective, hemostatic, and consciousness alterations related to the use of psychotropic medications, and drug–drug interactions between psychotropic and medical treatments used in people with COVID-19, was reviewed and discussed by the working group.

**Results:**

All classes of psychotropic medications showed potentially relevant safety risks for people with COVID-19. A set of practical recommendations was drawn in order to inform frontline clinicians on the assessment of the anticipated risk of psychotropic-related unfavorable events, and the possible actions to take in order to effectively manage this risk, such as when it is appropriate to avoid, withdraw, switch, or adjust the dose of the medication.

**Conclusions:**

The present evidence-based recommendations will improve the quality of psychiatric care in people with COVID-19, allowing an appropriate management of the medical condition without worsening the psychiatric condition and vice versa.

## Background

The novel coronavirus outbreak is a global health emergency calling for a rapid adaptation of conventional clinical practices in many medical areas, including psychiatry. Coronavirus disease (COVID-19) is a systemic infection potentially targeting multiple organs and functions. Interstitial pneumonia is the landmark feature of this condition, leading to severe respiratory distress requiring intensive life support in about one out of twenty symptomatic cases [1, 2]. Old age and pre-existing medical comorbidities are associated with increased severity and mortality [3].

Although there is debate about the efficacy and safety of medical treatments to prevent complications and decrease mortality [4], current clinical protocols generally include the off-label use of chloroquine, hydroxychloroquine, antiviral medications, anticoagulant prophylaxis, and immune system modulators (e.g., interferons) [5–7].

People with COVID-19 may frequently experience a new onset or exacerbation of psychiatric manifestations in response to the communication of the diagnosis, the need for forced isolation, the presence of severely distressing medical symptoms, and the possible risk of death. In addition, intensive care support and experimental medical treatments with psychiatric side effects (e.g., antimalarials) might be an additional risk factor for the onset psychiatric symptoms and altered states of consciousness, including delirium [8, 9]. Epidemiologic data, although preliminary, showed that up to one of four patients might experience symptoms of anxiety or depression [10] and about 15% might develop impaired consciousness states [11], which is likely to be associated with a remarkable increased risk of death [12].

For these reasons, people with COVID-19 may require treatment with medications targeting psychiatric manifestations. As in the general population these medications are associated with a wide range of safety concerns, in people with COVID-19, their use may be particularly challenging. Psychotropic medications may interact with the medical treatments for COVID-19, and some of their adverse effects may worsen the course and outcome of the underlying medical condition. In this context, the aim of this evidence review and practical recommendations is to make frontline doctors (including psychiatrists, other specialists, and general practitioners) aware of clinically relevant safety issues of psychotropic medication use in people with COVID-19 and possible management strategies.

## Methods

The process of evidence retrieval, appraisal, and discussion followed the methodology of the World Health Organization (WHO) Rapid Advice Guidelines in the context of a public health emergency [13]. Details of the process are reported in Additional File 1 [3, 10–97]. Results were reported following the AGREE statement [14]. A multi-disciplinary international working group was established ad hoc. Professionals with expertise in research methodology, guideline development, epidemiology, consultation-liaison psychiatry, and clinical psychopharmacology were involved (see Additional File 1: Table S1). Potentially relevant financial and intellectual interests were publicly disclosed in advance and independently assessed by all members, in order to minimize potential biases. On the basis of a shared process directly involving all members, the following key clinical elements were considered of utmost priority in terms of potential safety issues of psychotropic medications in people with COVID-19: (a) drug–drug interactions, (b) respiratory risk, (c) cardiovascular risk, (d) risk of infections, (e) coagulation risk, and (f) risk of delirium. The following classes of psychotropic medications, as defined by the Anatomical Therapeutic Chemical (ATC) classification system, were included: antidepressants, anxiolytics, antipsychotics, and selected antiepileptics employed for the treatment of mood disorders.

A literature search, last updated on the 8 May 2020, was conducted using PubMed, Web of Science Core Collection, and the Cochrane Database of Systematic Reviews. Terms describing psychotropic medications were combined with terms describing the key clinical elements identified. The search was limited to up-to-date systematic reviews published in the last 10 years. Priority was given to systematic reviews reporting a quantitative synthesis of safety outcomes of psychotropic medications in the general population or, if available, in people with medical conditions or vulnerabilities similar to those of COVID-19: respiratory diseases, cardiovascular diseases, and elderly patients. Both in- and outpatient settings were considered (see Additional File 1: Tables S2 and S3). The search output was screened and selected by one review author (DP) and independently checked for accuracy by two other review authors (GO, CB). Information on population, intervention, comparison, and outcomes of interest was extracted from the included systematic reviews, and a narrative synthesis was developed to support the working group discussion. Methodological quality of the included systematic reviews was assessed using AMSTAR-2 to aid interpretation of the results, while the certainty of evidence according to the GRADE methodology was not assessed.

Information on drug–drug pharmacokinetic and pharmacodynamic interactions between psychotropic medications and medical treatments for COVID-19 was searched by cross-checking four drug–drug interaction databases (namely the Food and Drug Administration (FDA) database, MediQ, PSIAC, and the COVID-19 database of the Liverpool University) [98–101] and the European Medicines Agency (EMA) and FDA-registered product characteristics [34, 102]. Additionally, PubMed was searched using the heading “drug interactions” combined with names of individual psychotropic medications and selecting only human studies performed in the last 5 years. When different sources provided a different estimate of the expected severity of an interaction, we used a conservative approach by reporting the most severe scenario. Evidence from pharmacokinetics simulations were not considered, as they might not apply to real-world patients [103]. The anti-COVID-19 medications considered included those routinely used off-label in current clinical practice protocols [6], and those currently undergoing rigorous experimental protocol, as reported by the WHO [7]. Drug–drug interactions were organized in a tabular layout and classified in four degrees of severity: (a) high risk, if a relevant clinical impact is likely as available evidence indicate strong inhibition or induction of the major metabolic pathways of medications, well-established adverse reactions related to drug–drug interactions, and if there are contraindications according to the package inserts; (b) moderate risk, if a relevant clinical impact is less likely according to the evidence, but cannot be excluded; (c) low risk, if a relevant clinical impact is unlikely according to the evidence; (d) very low risk, if the evidence indicate that no relevant clinical impact is expected.

All members of the working group individually reviewed the narrative synthesis of the literature and the tabular synthesis of drug–drug interactions, and subsequently, collegial discussions were organized electronically. The discussion was moderated in order to appraise the available evidence in light of possible values and preferences, clinical expertise considerations, certainty of the evidence retrieve, and feasibility issues, according to the GRADE Evidence-to-Decision Framework [104]. Following discussion, the working group formulated practical recommendations for clinicians. In case of disagreements, a vote was taken. Agreement by ≥ 80% of experts was required for a clinical statement to be retained. Considering the rapid process employed, no external review was performed.

## Results

The literature search provided 1531 hits. After duplicate removal and screening of title and abstract, 113 articles were retained for full inspection. Finally, 10 systematic reviews providing a quantitative synthesis on the outcomes of interest were selected, and 64 articles included as additional material (see Additional File 1: Fig. S1; Tables S4, S5 and S6). Data were extracted and synthesized by the scientific secretariat in order to inform the discussion of the working group. The extended version of the evidence synthesis is available in Additional File 1. Table 1 shows the included systematic reviews, the outcomes extracted, and their quality according to the AMSTAR-2 checklist (see also Additional File 1: Table S7). In terms of populations, we found relatively limited evidence on medical conditions comparable to COVID-19, while most evidence was on safety outcomes of psychotropic medications in the general population.

**Table 1:** Systematic reviews reporting a quantitative synthesis of the evidence for the outcomes selected by the working group

Review	Type of study	Population	Intervention	Comparison	Outcome	Summary	AMSTAR-2
Clegg et al. 2011 [23]	Systematic review of observational studies and RCTs (no meta-analysis)	Patients from medicine and surgery settings, mostly elderly	Antidepressants Antipsychotics Benzodiazepines	No exposure or placebo	Risk of delirium	Increased risk for **benzodiazepines** as a class (1 case–control study; *n* = 1341; OR 3.0, 95% CI 1.3 to 6.8), with higher risk for longer-acting agents and higher doses; **antipsychotics** as a class (1 prospective cohort study; *n* = 325; OR 4.5; 95% CI 1.8 to 10.5), but not for haloperidol (1 RCT; *n* = 430; OR 0.9, 95% CI 0.6 to 1.3); and **tricyclic antidepressants** (1 RCT; *n* = 111; RR 1.7, 95% CI 1.4 to 2.1).	Critically low
Dragioti et al. 2019 [30]	Umbrella review (meta-analyses of observational studies)	Mixed (general population; people with depression)	Antidepressants	No exposure	Cardiovascular risk	Increased risk of coronary heart disease for **TCAs** (*N* = 14; *n* = 347,750; OR 1.51, 95% CI 1.07 to 2.12; CE IV).	Critically low
						Risk of acute heart disease not increased for **SSRIs** (*N* = 14; *n* = 818,337; RR 1; 95% CI 0.83 to 1.22).	Critically low
						Risk of myocardial infarction not increased for **antidepressants** as a class (*N* = 21; *n* = 1,793,877; RR 1.03, 95% CI 0.88 to 1.22).	Critically low
						Increased risk of cerebrovascular disease for **SSRIs** (*N* = 6; *n* = 280,784; RR 1.26, 95% CI 1.14 to 1.39; CE III).	Critically low
						Risk of cerebrovascular disease not increased for **TCAs** (*N* = 4; *n* = 278,749; RR 1.06, 95% CI 0.96 to 1.17).	Critically low
					Coagulation risk	Increase risk of severe bleeding at any site for **SSRIs** and **SNRIs** taken together (*N* = 44; *n* = 1,443,029; OR 1.41, 95% CI 1.27–1.57; CE II).	Critically low
Dzahini et al. 2018 [32]	Systematic review and meta-analysis of observational studies	Mixed (general population; people with schizophrenia, bipolar disorder, depression), mostly elderly	Antipsychotics	No exposure	Risk of infections	Risk of pneumonia is increased by **FGAs** (*N* = 5; *n* = 29,510, RR 1.69, 95% CI 1.34 to 2.15), **SGAs** (*N* = 6; *n* = 30,656; RR 1.93, 95% CI 1.55 to 2.41) and **antipsychotics** as a class (*N* = 7; *n* = 30,760; RR 1.83, 95% CI 1.60 to 2.10). No differences emerged between FGAs and SGAs.	Critically low
Huhn et al. 2019 [48]	Systematic review and network meta-analysis of RCTs	Adults with multi-episode schizophrenia	Antipsychotics	Placebo	Cardiovascular risk	Significantly increased risk of QTc prolongation (*N* = 51; *n* = 15,467): quetiapine (OR 3.43, 95% CI 0.94 to 6.0); olanzapine (OR 4.29, 95% CI 1.91 to 6.68); risperidone (OR 4.77, 95% CI 2.68 to 6.87); iloperidone (OR 6.93, 95% CI 4.49 to 9.36); ziprasidone (OR 9.7, 95% CI 7.43 to 12.04); amisulpride (OR 14.1, 95% CI 7.71 to 20.45); serenditole (OR 23.9, 95% CI 20.56 to 27.33).	Low
Kunutsor et al. 2018 [55]	Systematic review and meta-analysis of observational studies	General population	Antidepressants	No exposure	Coagulation risk	Increased risk of venous thromboembolism for **antidepressants** as a class (*N* = 6; *n* = 828,327; OR 1.27; 95% CI 1.06 to 1.51), **TCAs** (*N* = 4; *n* = 59,161; OR 1.16; 95% CI 1.06 to 1.27), **SSRIs** (*N* = 4; *n* = 58,088; OR 1.12; 95% CI 1.02 to 1.23), and **other antidepressants** (*N* = 3; *n* = 3198; OR 1.59, 95% CI 1.21 to 2.09).	Critically low
Lu et al. 2016 [59]	Systematic review and meta-analysis of RCTs	Adults with insomnia and COPD	Benzodiazepines	Placebo	Respiratory risk	No differences between benzodiazepines (i.e., triazolam and temazepam) and placebo in terms of percentage of time below 90% arterial oxygen saturation during sleep (*N* = 3; *n* = 94; weighted MD 1.32; 95% CI − 7.33 to 9.97) and other respiratory outcomes during sleep (sleep apnea, Apnea–Hypopnea Index, arterial oxygen saturation).	Critically low
Ostuzzi et al. 2019 [105]	Systematic review and meta-analysis of RCTs	Adults with depression and ischemic heart disease	Antidepressants	Placebo	Cardiovascular risk	No differences emerged for antidepressants as a class (SSRIs the most represented) in terms of mortality because of cardiovascular events (*N* = 14; *n* = 2674; RD 0.0, 95% CI − 0.01 to 0.01) and nonfatal cardiac events (*N* = 9; *n* = 1869; RD − 0.01, 95% CI − 0.04 to 0.02).	High
Papola et al. 2019 [70]	Umbrella review (meta-analysis of observational studies)	Mixed (general population; people with dementia, schizophrenia, or other psychiatric conditions), mostly elderly	Antipsychotics	No exposure	Cardiovascular risk	For antipsychotics as a class, there was an increased risk of sudden cardiac death (*N* = 6; *n* = 677,488; OR 2.24; 95% CI 1.45 to 3.46; CE III), myocardial infarction (*N* = 9; *n* = 399,868; OR 2.21; 95% CI 1.41 to 3.46; CE III), and stroke (*N* = 9; *n* = 65,700; OR 1.45, 95% CI 1.24 to 1.7; CE III). Meta-regression showed that the risk of myocardial infarction and stroke was higher in the elderly.	Moderate
					Coagulation risk	For antipsychotics as a class, there was an increased risk of venous thromboembolism (*N* = 14; *n* = 31,417,175; OR 1.55, 95% CI 1.31 to 1.83; CE II).	Low
Pollok et al. 2018 [72]	Systematic review and meta-analysis of RCTs	Adults with depression and COPD	Antidepressants	Placebo	Respiratory risk	No increased risk of respiratory impairment for **TCAs** (change in dyspnea during walk according to the Pulmonary Functional Status Instrument: *N* = 1; *n* = 30; MD 0.50; 95% CI − 1.34 to 2.34) and **SSRIs** (change in FEV_1_: *N* = 2; *n* = 148; MD 0.01; 95% CI − 0.03 to 0.05).	High
Schneider-Thoma et al. 2019 [78]	Systematic review and meta-analysis of RCTs	Mixed (83% adults; 8% elderly; 42% schizophrenia; 30% bipolar disorder; 11% depression; 6% dementia)	Antipsychotics (mostly second-generation)	Placebo	Respiratory risk	Increased risk of respiratory, thoracic, and mediastinal serious adverse events in studies with at least one serious adverse event according to a maximum estimate (worst-case scenario) (*N* = 38; *n* = 13,007; OR 1.72; 95% CI 1.02 to 2.89).	Low
					Cardiovascular risk	Risk of cardiac (*N* = 54; *n* = 19,642; OR 1.22; 95% CI 0.85 to 1.75) and vascular serious adverse events (*N* = 33; *n* = 12,842; OR 1.82; 95% CI 0.97 to 3.41) were not increased in studies with at least one serious adverse event according to a maximum estimate (worst-case scenario).	Low
					Risk of infections	Increased risk of infections in studies with at least one serious adverse event according to a maximum estimate (worst-case scenario) (*N* = 88; *n* = 28,479; OR 1.43; 95% CI 1.06 to 1.92).	Low
Sun et al. 2018 [83]	Systematic review and meta-analysis of observational studies	General population, mostly elderly	Benzodiazepines	No exposure	Risk of infections	Increased risk of pneumonia for benzodiazepines and related medications (e.g., zolpidem) (*N* = 10; *n* = 1,520,285; OR 1.25; 95% CI 1.09 to 1.44). The risk was confirmed for current and recent exposure (not for past exposure); for long-acting, intermediate-acting and short-acting agents; and for younger and older patients.	Critically low
Wu et al. 2019 [95]	Systematic review and network meta-analysis of RCTs	Medical and surgical patients at risk of delirium	Antipsychotics Benzodiazepines Mood stabilizers	Placebo/treatment as usual	Risk of delirium	Decreased incidence of delirium as compared to placebo or treatment as usual (*N* = 38; *n* = 8168) emerged for **olanzapine** (OR 0.25; 95% CI 0.09 to 0.69) and **risperidone** (OR 0.27; 95% 0.07 to 0.99), while no differences emerged for lorazepam, haloperidol, and gabapentin. A higher incidence emerged for **midazolam hydrochloride** (OR 2.98; 95% CI 1.30 to 6.80).	Low

*AD* antidepressant, *AP* antipsychotic, *CE* credibility-of-evidence classification (I = convincing evidence; II = highly suggestive evidence; III = suggestive evidence; IV = weak evidence), *CI* confidence interval, *FEV* forced expiratory volume, *FGA* first-generation antipsychotic, *ICU* intensive care unit, *MA* meta-analysis, *MD* mean difference, *N* number of studies included in the analysis, *n* number of participants included in the analysis, *OR* odds ratio, *RCT* randomized controlled trial, *SGA* second-generation antipsychotic, *SR* systematic review, *RR* risk ratio, *SNRI* serotonin–norepinephrine reuptake inhibitors, *SSRI* selective serotonin reuptake inhibitor, *TCA* tricyclic antidepressant, *VTE* venous thromboembolism

### Synthesis of the evidence

#### Drug–drug interactions

In patients with COVID-19, the risks of drug–drug interactions involving psychotropic medications might be relevant. Firstly, the bioavailability and disposition of several psychotropic medications might be importantly affected by COVID-19-related systemic inflammation processes [65], impaired liver functioning [35], and abrupt smoking cessation [45, 46, 64]. Secondly, psychotropic medications and medical treatments can reciprocally affect each other’s plasma levels by inducing or inhibiting cytochrome P450 (CYP) activity to an extent which is poorly understood and hardly predictable [37]. Thirdly, these combinations are at risk of pharmacodynamic interactions, and particularly QTc prolongation, immunity, and coagulation abnormalities. Pharmacokinetic and pharmacodynamic interactions for a selection of psychotropic medications, and indications for their management, are synthetically reported in Table 2, while a detailed table extensively reporting all psychotropic medications is available in Additional File 1: Table S8.

**Table 2:**
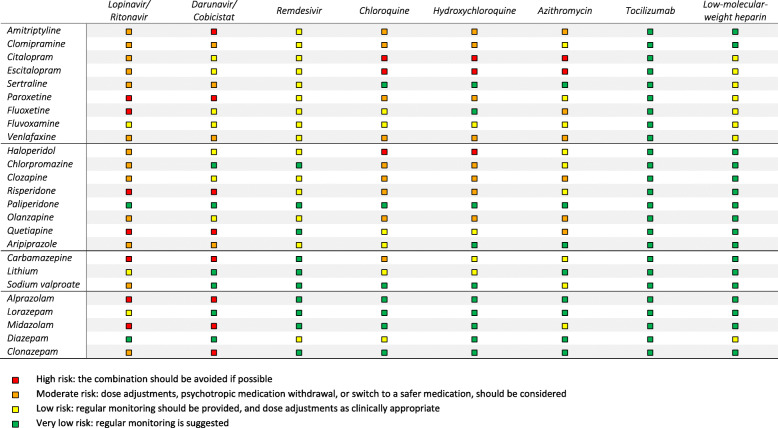
Clinical risk and actions recommended for selected drug–drug interactions between psychotropic and medical treatments for COVID-19

#### Respiratory risk

COVID-19-related bilateral interstitial pneumonia is associated with hypoxic respiratory distress and can rapidly evolve into a full-blown acute respiratory distress syndrome (ARDS) [106], which is the major cause of death in people with COVID-19 [29, 106].

Data from randomized trials on antidepressants did not show an increased risk of respiratory distress and overall mortality in patients with COPD (including elderly patients) exposed to selective serotonin reuptake inhibitors (SSRIs) and tricyclic antidepressants (TCAs) [72] and authoritative guidelines indicate SSRIs as a safe choice in people with medical conditions (including respiratory disease) [67]. However, data from a recent, large observational study showed a higher risk for COPD worsening or COPD-related hospitalization and mortality in older patients taking SSRIs and SNRIs versus those not exposed [89].

Antipsychotics are associated with an increased risk of respiratory, thoracic, and mediastinal serious adverse events according to data from randomized trials [78] (Table 1). The risk of respiratory distress is probably higher for highly sedative agents, particularly at higher doses, in combination, and when they are prescribed in patients with pre-existing respiratory impairment [39, 90]. In case of psychomotor agitation requiring rapid tranquilization with antipsychotics (e.g., hyperkinetic delirium), the risk for acute extrapyramidal symptoms (e.g., dystonia, with possible impaired swallowing and consequent risk of aspiration) and reduced mobility can notably worsen respiratory distress [80, 107].

Mood stabilizers have mild-to-moderate sedative profiles, and there is no evidence of a relevant risk for excessive sedation and related respiratory distress [24, 66].

Although the risk of respiratory suppression with benzodiazepines is notably lower than barbiturates or other neuromuscular blocking agents [21, 50, 52], it may be relevantly high in people with acute respiratory distress and in the elderly [33, 42, 80, 88]. The risk of respiratory distress is related to the differential sedative properties of different agents, their half-life, and is usually dose-dependent [33, 41, 49, 88]. Data from randomized trials showed no relevant impact on respiratory outcomes in people with chronic obstructive pulmonary disease (COPD) treated with benzodiazepines for insomnia, although the pooled sample size was relatively small (Table 1) [59].

#### Cardiovascular risk

People with COVID-19 may have several cardiovascular risk factors, including (a) old age; (b) pre-existing comorbid cardiovascular diseases; (c) use of medical treatments with QTc-prolonging properties, often in combination (e.g., antivirals, chloroquine/hydroxychloroquine and antibiotics); (d) a possible direct cardiotoxic effects of the coronavirus; and (e) electrolyte alterations related to abnormal respiratory gas exchange [3, 20, 87, 92, 97]. The most important risk factors of severe arrhythmias, such as torsade de pointes, include the magnitude of QTc prolongation, pre-existing heart disease, female sex, bradycardia, hypokalemia, and other electrolyte abnormalities [44].

Data from randomized studies in people with ischemic heart disease did not show an increased risk of cardiovascular mortality and nonfatal cardiac events for antidepressants (particularly SSRIs) [105]. On the other hand, data from observational studies showed an increased risk of coronary heart disease for tricyclic antidepressants (TCAs), but not SSRIs and antidepressants as a class, while SSRIs but not TCAs were associated with an increased risk for cerebrovascular disease [30] (Table 1). Tricyclic antidepressants and, to a lesser extent, citalopram, escitalopram, and venlafaxine have been associated with QTc prolongation, with a possibly higher risk in older patients [44, 75].

Antipsychotics have been shown to be associated with serious cardiovascular events according to data from observational studies assessing sudden cardiac death, myocardial infarction, and stroke [70], while data from randomized trials confirmed an increased risk of QTc prolongation for a number of antipsychotics [48], but not a higher risk of serious cardiac and vascular adverse events [78] (Table 1). Antipsychotic combination and higher cumulative doses might contribute to QTc prolongation [18, 85]. The differential risk of QTc prolongation of antipsychotics is not entirely consistent across different data sources and study designs [44, 48]. In general, the risk of QTc prolongation should not be neglected for any antipsychotic [44, 71], although its predictive proprieties on TdP are still unclear [79].

The risk of arrhythmias is probably very low for mood stabilizers and benzodiazepines [50, 66], with the possible exception of lithium, for which benign electrocardiographic changes and cases of ventricular arrhythmia and sudden cardiac death have been described [34, 61].

#### Risk of infections

Systemic dysregulation of immunity and inflammation response is a key feature of COVID-19. The severity of inflammatory parameters (such as IL-6) has been associated with the fatality risk [62, 74], and immunosuppressive therapies may play a role in treatment and prevention of complications [63].

Antidepressants have been consistently shown to have anti-inflammatory proprieties, although little is known about their possible role in systemic infections [38, 54, 84]. In vitro studies showed a protective effect against bacteria and fungi [26], but clinical data are unclear, as a possibly higher risk of *Clostridium difficile* infection has been reported [47]. Tricyclic antidepressants, and particularly clomipramine and imipramine, have been associated with possible blood dyscrasias, including neutropenia [58].

Antipsychotics have been associated with immunosuppressive proprieties, such as decreased pro-inflammatory cytokine levels, blood dyscrasias, and altered production of antibodies [60, 73, 76, 77]. The risk of neutropenia is about 1% for clozapine (3% in the elderly) and 0.1% for phenothiazines [36], while for other medications data are sparse [68]. Furthermore, both first- and second-generation antipsychotics have been associated with a higher risk of pneumonia in observational studies [32]. Data from randomized trials including mostly second-generation antipsychotics showed a higher risk of infections [78] (Table 1). Apart from immunity abnormalities [82], multiple mechanisms may contribute, including reduced clearance of the airways (related to central sedation and inhibition of cough), impaired chest movements and swallowing due to extrapyramidal symptoms, and sialorrea [51]. This risk might be particularly relevant for clozapine [27].

Carbamazepine, oxcarbazepine, and, to a lesser extent, sodium valproate, have been associated with an increased risk of neutropenia, while lithium appears to be free from relevant immunological effects [66].

Data from observational studies showed an increased risk of pneumonia for benzodiazepines as compared to non-users for both older and younger patients, short-term and long-term use, short- and long-term acting agents, and current and recent users [83] (Table 1).

#### Coagulation risk

Blood hypercoagulability related to inflammatory endothelial dysfunction has been largely reported in patients with COVID-19, ranging from mild manifestations to life-threatening conditions, such as disseminated intravascular coagulation [29, 31]. Low molecular weight heparin has been suggested as an effective prophylaxis since early stages of the disease [22].

Antidepressants have been associated with various hemostasis alterations [43]. Observational studies shown an increased risk of severe bleeding at different sites has been shown for SSRIs and serotonin–norepinephrine reuptake inhibitors (SNRIs) [30] and an increased risk of thromboembolism for all antidepressant classes [55]. The risk of bleeding is arguably higher in vulnerable patients (e.g., old age, pre-existing coagulation abnormalities, anticoagulant therapy, major surgery) [16, 57].

Antipsychotics have been clearly shown to be associated with an increased risk of thromboembolism in large observational studies, with an arguably higher risk in vulnerable populations with pre-existing risk factors [70] (Table 1). It is still uncertain if there are relevant differences in risk between individual agents [108].

The risk for pro- or anticoagulant effect is likely to be low for mood stabilizers and benzodiazepines [34].

#### Risk of delirium

Although epidemiological data are preliminary, delirium has been frequently described in people with COVID-19 [11] and is associated with unfavorable prognosis [12]. Old age, medical comorbidities, dementia, and multiple pharmacological treatments are well-known risk factors for both delirium and COVID-19 severity [3, 91]. Neurotropic mechanisms of COVID-19 have been also hypothesized [17]. Furthermore, many of the experimental medical treatments use for COVID-19 have a well-known risk for neuropsychiatric side effects (e.g., antimalarial and antiviral medications, interferons, corticosteroids) and may represent an additional risk. Some psychotropic medications are also known as risk factors for delirium. In particular, benzodiazepines, antidepressants with anticholinergic proprieties (mainly TCAs, but possibly also paroxetine), and lithium are considered at high risk according to data from observational studies [23] (Table 1). Anticholinergic medications are often a precipitating factor and are associated with delirium severity. It has been estimated that medications alone might account for up to 40% of cases of delirium [15, 23]. Data from a recent meta-analysis of randomized trials showed that olanzapine and risperidone were effective in preventing delirium as compared to placebo or treatment as usual, while midazolam increased its incidence [95] (Table 1).

### Evidence-based practical recommendations

Based on the considerations reported above and after collegial discussion, and taking into consideration values, feasibility, resource use, and certainty of the evidence according to the Evidence-to-Decision framework (see Additional File 1: Table S9), the following practical recommendations were formulated:

1. The risk and severity of drug–drug pharmacokinetic and pharmacodynamic interactions between COVID-19 medical treatments and psychotropic medications should always be assessed, taking into account the additional vulnerability related to the underlying medical condition (e.g., cardiovascular conditions increasing the risk of QTc prolongation).
2. In case of high-risk interactions, the combination should be avoided if possible. In case of moderate-risk interactions, dose adjustments, psychotropic medication withdrawal, or switch to a safer medication should be considered. In case of low-risk interactions, regular monitoring should be provided, with dose adjustments as clinically appropriate. In case of very low-risk interaction, regular monitoring is suggested (see Table 2 and Additional File 1: Table S8).
3. An estimation of psychotropic-related risk of respiratory depression should systematically take into account the following: (a) the intrinsic sedative proprieties of psychotropic medications, their half-life (higher risk for longer half-life), the dose, and the occurrence of other aspects possibly impairing respiration (e.g., reduced motility, sialorrhea); (b) pharmacokinetic interactions raising plasma levels of sedative medications (e.g., lopinavir/ritonavir combined with quetiapine) and pharmacodynamic interactions (e.g., co-treatments with opioids); and (c) pre-existing respiratory impairment (e.g., COPD) and degree of COVID-19-related respiratory depression.
4. Antipsychotic medications are at risk of worsening respiratory function in people with COVID-19, particularly at high doses and when used in combination. Antipsychotics with highly sedative profiles should be avoided or used short term.
5. The risk of respiratory impairment associated with benzodiazepines in the general population is debated, but might be particularly relevant in elderly patients with COVID-19 and pre-existing comorbidities (e.g., COPD). Benzodiazepines should be avoided or used short term (e.g., control of acute agitation), preferring those with shorter half-life (e.g., etizolam, oxazepam, lorazepam). Although antidepressants are generally considered safe in terms of respiratory impairment, caution is advised as data are controversial.
6. An estimation of psychotropic-related risk of cardiovascular events should systematically take into account the following: (a) the intrinsic QTc-prolonging proprieties of psychotropic medications, their cumulative dose, and use in combination; (b) pharmacokinetic interactions possibly raising plasma levels of QTc-prolonging medications and pharmacodynamic interactions (e.g., co-treatments with antivirals, chloroquine, hydroxychloroquine, and opioids); and (c) pre-existing cardiovascular conditions (in particular, ischemic heart disease) and COVID-19-related cardiovascular conditions.
7. For interactions with low-to-moderate risk of QTc prolongation, an adjustment towards a lower dose of one or both medications is generally required, along with regular electrocardiogram monitoring. In case these interactions add up with other risk factors for QTc prolongation (e.g., cardiovascular comorbidities, electrolyte abnormalities), medications at risk should be avoided, or withdrawn, or switched to safer medications, as clinically appropriate.
8. Antipsychotics, benzodiazepines, and some mood stabilizers may be associated with an increased risk of secondary infections in people with COVID-19, and possibly with an unfavorable course of systemic infections. The risk is likely to be particularly relevant for clozapine, carbamazepine, and oxcarbazepine. Regular monitoring is therefore indicated.
9. In people with COVID-19, both antipsychotics and antidepressants might increase the risk of thromboembolism, particularly in the elderly. In people with COVID-19 taking heparin prophylaxis, antidepressants might increase the risk of bleeding, with a higher risk for serotoninergic agents (i.e., SSRIs and SNRIs), especially in elderly patients. Regular monitoring is indicated. In case there are additional risk factors for bleeding (e.g., other coagulation abnormalities, old age, anticoagulant therapy, major surgery), a dose adjustment or withdrawing the medications at risk should considered, as clinically appropriate.
10. In people with COVID-19 and known risk factors for delirium (e.g., old age, dementia, multiple comorbidities), the use of agents with anticholinergic properties (e.g., tricyclic antidepressants and paroxetine), benzodiazepines (particularly midazolam), and lithium should generally be avoided.
11. In patients with COVID-19 who are already in treatment with psychotropic medications, an accurate assessment of current psychiatric symptoms and past psychiatric history is important in order to review the need of continuing treatment and its dose.
12. In addition to psychotropic medications, and when drug treatment is clinically inappropriate, clinicians should carefully assess whether adequate supportive psychosocial interventions are provided, including electronically delivered interventions.

## Discussion

### Implications for practice

The dramatic situation created by the COVID-19 pandemic requires rapid adjustments to the dynamic interplay between medical and psychiatric needs of patients. This literature review explored a number of safety issues relevant for the management of psychotropic medications in people with COVID-19 and informed the discussion of a working group of clinical and research experts.

In general, we found that all classes of psychotropic medications have potentially relevant safety issues for people with COVID-19. The magnitude of risk of individual agents or classes of medications was unclear or unreliable in most cases, considering the paucity of data, and the relevant indirectness of populations considered. Unavoidably, in clinical practice, the risk of unfavorable outcomes needs to be carefully weighed on a case-by-case basis, in light of a number of co-existing risk factors. It is therefore challenging to provide recommendations limited to specific clinical situations or single medications. Moreover, although different safety issues have been explored separately, they are actually broadly overlapping (i.e., respiratory function might be impaired by both the sedative effect of medications and the increased risk for respiratory infections).

Although the working group selected a number of safety issues to address, other principles of drug management should not be overlooked. In particular, as acute multifactorial hepatic and kidney injury has been described in people with COVID-19 [109, 110], liver and kidney functioning should be closely monitored. Possibly hepatotoxic (e.g., valproate, carbamazepine, tricyclic antidepressants) and nephrotoxic psychotropic medications (e.g., lithium), as well as psychotropic medications extensively metabolized by the liver (such as most of antidepressants, antipsychotics, and mood stabilizers) and subject to renal excretion (e.g., lithium, gabapentin, topiramate, pregabalin, and paliperidone), should be routinely revised in order to adjust the dose or withdraw the medication in case of high clinical risk.

Practical recommendations were formulated in order to support clinicians in the assessment and management of the risk related to psychotropic medications. In many cases, adjusting the dose of medical or psychotropic medications is probably a satisfactory and pragmatic safety measure. However, when the risk of severe adverse events is relevant, withdrawing the medication or switching to a safer one might be required. In any case, an accurate assessment of current psychopathology is key, considering that, for some patients, psychotropic treatments are essential (e.g., long-standing maintenance with antipsychotics or mood stabilizers) and should be safeguarded, while, for some other patients, medications can be decreased in dose or even withdrawn (considering for example that benzodiazepines and antidepressants are frequently prescribed inappropriately) [111, 112], provided that good practices for managing withdrawal risk are followed [56, 94]. As a general consideration, the working group agreed that supportive psychosocial interventions (even electronically delivered and provided by non-specialist health-care providers) [113–115] should not be neglected in order to mitigate the emotional stress and pressure that can exacerbate both psychiatric and medical conditions [116], and this is particularly relevant when pharmacological interventions are limited or unfeasible. Arguably, this set of simple recommendations can be easily applicable in clinical practice, as no particular limitations emerged in terms of costs, as well as values and preferences of patients and key stakeholders (see Additional File 1: Table S9), and the principles described are easily accessible not only for psychiatrists, but also for other specialists directly involved in the care of people with COVID-19.

### Limitations

Several limitations should be acknowledged. Firstly, following the WHO Rapid Advice Guidelines for public health emergencies [13], a simplified methodology was employed for evidence gathering and aggregation, including the lack of a review protocol, a simplified search process (limitation to articles published in the last 10 years, no clear-cut predefined inclusion and exclusion criteria), the lack of a formal assessment of the certainty of evidence with the GRADE methodology, the lack of external review of the process, and the lack of indications for a process of audit and feedback (see Additional File 1: Table S10). Furthermore, the working group included mostly psychiatrists and experts in research methodology, while other potentially interested stakeholders were not involved. Secondly, the working group decided to give priority to safety issues of relevance for both psychiatrists, infective disease doctors, and other specialists, while the efficacy of psychotropic medications in people with severe medical illness was not assessed. Thirdly, in many cases, the clinical relevance of drug–drug interactions was difficult to ascertain, considering both the scarcity of data and the multitude of potential co-occurring factors possibly influencing the metabolism, distribution, and target action of medical and psychotropic medications. Fourthly, all medical treatments for COVID-19 are currently employed off-label according to principles of compassionate use. The list of medications included in this review cannot be considered exhaustive, as it is possible that new treatments will undergo research scrutiny and be employed in clinical practice, as the field is rapidly evolving. In this case, the working group will update the search of the evidence and content of recommendations if needed. Also, considering the global threat represented by COVID-19 and the novelty of this condition, the search for rapid solutions might lead to an uncontrolled use of off-label medications [117]. This problem is likely to involve also psychotropic medications, considering that the use of standard treatments might be limited due to the underlying complex medical conditions. A final limitation is that the role of psychosocial interventions in the optimal management of psychotropic drugs was not formally addressed with a dedicated search and discussion.

## Conclusions

Currently, many patients with COVID-19 require treatment with psychotropic medications, whose appropriate management is particularly challenging in light of the underlying medical condition and the high risk of drug–drug interactions. Clinicians need to be vigilant when initiating psychotropic agents in patients receiving medical drugs for COVID-19. Similarly, when deciding to prescribe experimental medical drugs in patients under long-term psychopharmacological treatment, clinicians need to be extremely cautious considering that medical treatments for COVID-19 are still experimental and their efficacy debated.

Hopefully, as clinical interventions can best be delivered when clear, evidence-based guidance is provided, the pragmatic principles described here can favor an optimal management of psychotropic medicines for patients with COVID-19, aiming to address potentially emerging psychopathology, maintain control of underlying psychiatric condition, mitigate the potentially aggravating effects of psychological stress, and, in general, manage the medical condition without worsening the psychiatric condition and vice versa.

## Supplementary information

**Additional file 1: Table S1**. Working Group composition. **Table S2**. PICO question and framework. **Table S3**. Search strategy. **Fig. S1**. PRISMA flow-chart. **Table S4**. List of included studies. **Table S5**. Additional material that informed the working group. **Table S6**. List of excluded studies, with reason. **Table S7**. AMSTAR-2 of included systematic reviews. **Table S8**. Drug-drug interactions table. Narrative synthesis of the evidence. **Table S9**. Evidence to decision framework. **Recommendations. Table S10**. AGREE Reporting Checklist.

## Data Availability

All data and materials that supported the discussion of the working group are reported in Additional File 1: Tables S4 to S9.

## Abbreviations

ATC: Anatomical Therapeutic Chemical
COPD: Chronic obstructive pulmonary disease
COVID-19: Coronavirus disease
CYP: Cytochrome P450
EMA: European Medicines Agency
FDA: Food and Drug Administration
SNRIs: Serotonin–norepinephrine reuptake inhibitors
SSRIs: Selective serotonin reuptake inhibitors
TCAs: Tricyclic antidepressants
WHO: World Health Organization
