# Correction to: Safety of psychotropic medications in people with COVID-19: evidence review and practical recommendations

**DOI:** 10.1186/s12916-020-01757-w

**Published:** 2020-09-12

**Authors:** Giovanni Ostuzzi, Davide Papola, Chiara Gastaldon, Georgios Schoretsanitis, Federico Bertolini, Francesco Amaddeo, Alessandro Cuomo, Robin Emsley, Andrea Fagiolini, Giuseppe Imperadore, Taishiro Kishimoto, Giulia Michencigh, Michela Nosé, Marianna Purgato, Serdar Dursun, Brendon Stubbs, David Taylor, Graham Thornicroft, Philip B. Ward, Christoph Hiemke, Christoph U. Correll, Corrado Barbui

**Affiliations:** 1 WHO Collaborating Centre for Research and Training in Mental Health and Service Evaluation, Department of Neuroscience, Biomedicine and Movement Sciences, Section of Psychiatry, University of Verona, Verona, Italy; 2 Department of Psychiatry, The Zucker Hillside Hospital, Northwell Health, Glen Oaks, NY USA; 3 Department of Molecular Medicine, University of Siena, Siena, Italy; 4 Department of Psychiatry, Faculty of Medicine and Health Sciences, Stellenbosch University, Tygerberg Campus, Cape Town, 8000 South Africa; 5 Azienda ULSS 9 Scaligera, Verona, Italy; 6 Department of Neuropsychiatry, Keio University School of Medicine, Tokyo, Japan; 7 Department of Psychiatry, University of Alberta, Edmonton, Alberta Canada; 8 Department of Psychological Medicine, Institute of Psychiatry, Psychology, and Neuroscience, King’s College London, London, UK; 9 Physiotherapy Department, South London and Maudsley National Health Services Foundation Trust, London, UK; 10 Pharmacy Department, Maudsley Hospital, London, UK; 11 Centre for Global Mental Health and Centre for Implementation Science, Institute of Psychiatry, Psychology and Neuroscience, King’s College London, London, UK; 12 School of Psychiatry, UNSW Sydney and Schizophrenia Research Unit, Ingham Institute of Applied Medical Research, Liverpool, NSW Australia; 13 Department of Psychiatry and Psychotherapy, University Medical Center of Mainz, Mainz, Germany; 14 Department of Psychiatry and Molecular Medicine, Zucker School of Medicine at Hofstra/Northwell, Hempstead, NY USA; 15 Department of Child and Adolescent Psychiatry, Charité Universitätsmedizin Berlin, Berlin, Germany

**Correction to: BMC Medicine 18, 215 (2020)**

**https://doi.org/10.1186/s12916-020-01685-9**

The original version of this article [1] unfortunately included an error to an author’s name. Author Serdar Dursun was erroneously presented as Dursun Serdar.

The author name has been updated in the original article and included in the author list of this Correction.
