# Supplementary material for: Safety of psychotropic medications in people with COVID-19: evidence review and practical recommendations
Source: BMC Med. 2020 Jul 15;18:215. doi: 10.1186/s12916-020-01685-9 (PMC7360478; doi:10.1186/s12916-020-01685-9)
Supplement: Supplementary file 1 — Additional file 1: Table S1. Working Group composition. Table S2. PICO question and framework. Table S3. Search strategy. Fig. S1. PRISMA flow-chart. Table S4. List of included studies. Table S5. Additional material that informed the working group. Table S6. List of excluded studies, with reason. Table S7. AMSTAR-2 of included systematic reviews. Table S8. Drug-drug interactions table. Narrative synthesis of the evidence. Table S9. Evidence to decision framework. Recommendations. Table S10. AGREE Reporting Checklist. [file 12916_2020_1685_MOESM1_ESM.docx]

**Safety of psychotropic medications in people with COVID-19: evidence review and practical recommendations**

Ostuzzi et al.

**Additional File 1**

**INDEX**

1. Table S1. Working Group composition
2. Table S2. PICO question and framework
3. Table S3. Search strategy
4. Figure S1. PRISMA flow-chart
5. Table S4. List of included studies
6. Table S5. Additional material that informed the working group
7. Table S6. List of excluded studies, with reason
8. Table S7. AMSTAR-2 of included systematic reviews
9. Table S8. Drug-drug interactions table
10. Narrative synthesis of the evidence
11. Table S9. Evidence to decision framework
12. Recommendations
13. Table S10. AGREE Reporting Checklist

**Table S1. Working Group composition**

| **Name, title** | **Affiliation** | **Area of expertise/contribution provided to the working group** |
| --- | --- | --- |
| Giovanni Ostuzzi, MD, PhD. Fellow researcher in Psychiatry | WHO Collaborating Centre for Research and Training in Mental Health and Service Evaluation, Department of Neuroscience, Biomedicine and Movement Science, Section of Psychiatry, University of Verona, Verona, Italy | - Consultation-liaison psychiatry; - Clinical psychopharmacology; - Pharmacoepidemiology; - Guidelines development methodology. |
| Davide Papola, MD. PhD candidate in Psychiatry | WHO Collaborating Centre for Research and Training in Mental Health and Service Evaluation, Department of Neuroscience, Biomedicine and Movement Science, Section of Psychiatry, University of Verona, Verona, Italy | - Guidelines development methodology; - Methodology of systematic reviews and meta-analysis; - Clinical psychopharmacology; - Database search and construction. |
| Chiara Gastaldon, MD. PhD candidate in Psychiatry | WHO Collaborating Centre for Research and Training in Mental Health and Service Evaluation, Department of Neuroscience, Biomedicine and Movement Science, Section of Psychiatry, University of Verona, Verona, Italy | - Pharmacoepidemiology; - Drug-drug interactions; - Methodology of systematic reviews and meta-analysis. |
| Giulia Michencigh, MD. Resident in Psychiatry | WHO Collaborating Centre for Research and Training in Mental Health and Service Evaluation, Department of Neuroscience, Biomedicine and Movement Science, Section of Psychiatry, University of Verona, Verona, Italy | - Consultation-liaison psychiatry; - Community psychiatry. |
| Marianna Purgato, PhD. Fellow researcher in Clinical Psychology | WHO Collaborating Centre for Research and Training in Mental Health and Service Evaluation, Department of Neuroscience, Biomedicine and Movement Science, Section of Psychiatry, University of Verona, Verona, Italy | - Clinical psychology; - Psychosocial interventions in medically ill patients; - Global mental health. |
| Michela Nosè, MD, PhD. Clincial Psychiatrist | WHO Collaborating Centre for Research and Training in Mental Health and Service Evaluation, Department of Neuroscience, Biomedicine and Movement Science, Section of Psychiatry, University of Verona, Verona, Italy | - Community psychiatry; - Global mental health; - Mental health care in refugees and asylum seekers; - Methodology of systematic reviews and meta-analysis. |
| Francesco Amaddeo, MD, PhD. Professor of Psychiatry | WHO Collaborating Centre for Research and Training in Mental Health and Service Evaluation, Department of Neuroscience, Biomedicine and Movement Science, Section of Psychiatry, University of Verona, Verona, Italy | - Consultation-liaison psychiatry; - Clinical epidemiology; - Iniquities in mental health. |
| Alessandro Cuomo, MD. PhD candidate in Psychiatry | Department of Molecular Medicine; University of Siena, Siena, Italy | - Clinical Psychopharmacology; - Personalised pharmacological algorhytms. |
| Robin Emsley, MD, PhD. Professor of Psychiatry | Department of Psychiatry, Faculty of Medicine and Health Sciences, Stellenbosch University, Cape Town, South Africa | - Clinical Psychopharmacology; - Psychopathology. |
| Andrea Fagiolini, MD. Professor of psychiatry | Department of Molecular Medicine; University of Siena, Siena, Italy | - Clinical Psychopharmacology; - Pharmacoepidemiology; - Personalised pharmacological algorhytms. |
| Giuseppe Imperadore, MD. Clincial Psychiatrist | ULSS 9 Scaligera, Verona | - Clinical Psychopharmacology; - Mental Health Services. |
| Taishiro Kishimoto, MD, PhD. Assistant Professor of Psychiatry | Keio University School of Medicine, Department of Neuropsychiatry, Tokyo, Japan | - Clinical psychopharmacology; - Neuropsychiatry. |
| Georgios Schoretsanitis, MD, PhD. Posdoctoral fellow in Psychiatry. | The Zucker Hillside Hospital, Department of Psychiatry, Northwell Health, Glen Oaks, NY, USA | - Drug-drug interactions; - Pharmacokinetics of psychotropic drugs; - Pharmacovigilance. |
| Dursun Serdar, MD, PhD. Professor of Psychiatry | Department of Psychiatry, University of Alberta, Edmonton, Alberta, Canada | - Biological psychiatry; - Pharmaco-Magnetic Resonance Imaging. |
| Brendon Stubbs, PhD. Physiotherapist, clinical lecturer | Department of Psychological Medicine, Institute of Psychiatry, Psychology, and Neuroscience, King's College London, London, UK;  Physiotherapy Department, South London and Maudsley National Health Services Foundation Trust, London, UK | - Non-pharmacological interventions in mental health; - Physical activity and mental health; - Physical rehabilitation. |
| David Taylor, MSc, PhD. Professor of Psychopharmacology | Pharmacy Department, Maudsley Hospital, London, UK | - Psychopharmacology; - Drug-drug interactions. |
| Graham Thornicroft, MD, PhD. Professor of Psychiatry | Centre for Global Mental Health and Centre for Implementation Science, Institute of Psychiatry, Psychology and Neuroscience, King’s College London, UK | - Social psychiatry; - Global mental health - Stigma and discrimination; - Implementation Science. |
| Philip Ward, MD, PhD. Professor of Psychiatry | School of Psychiatry, UNSW Sydney and Schizophrenia Research Unit, Ingham Institute of Applied Medical Research, Liverpool, NSW, Australia | - Biological psychiatry; - Brain imaging; - Psychopharmacology. |
| Christoph Hiemke, PhD. Professor of Clinical Pharmacology | Department of Psychiatry and Psychotherapy, University Medical Center of Mainz, Germany | - Drug-drug interactions; - Pharmacokinetics of psychotropic drugs; - Pharmacovigilance. |
| Cristoph U. Correll, MD. Professor of Psychiatry | The Zucker Hillside Hospital, Department of Psychiatry, Northwell Health, Glen Oaks, NY, USA;  Zucker School of Medicine at Hofstra/Northwell, Department of Psychiatry and Molecular Medicine, Hempstead, NY, USA;  Charité Universitätsmedizin Berlin, Department of Child and Adolescent Psychiatry, Berlin, Germany | - Clinical psychopharmacology; - Risk–benefit evaluation of psychotropic medications; - Clinical epidemiology. |
| Corrado Barbui, MD. Professor of Psychiatry | WHO Collaborating Centre for Research and Training in Mental Health and Service Evaluation, Department of Neuroscience, Biomedicine and Movement Science, Section of Psychiatry, University of Verona, Verona, Italy | - Clinical epidemiology; - Pharmacoepidemiology; - Global mental health; - Guidelines development methodology. |

Based on the assessment of the competing interests declared, all members of the working group took part to the discussion and voting.

**Tale S2. PICO question and framework**

**PICO question:** Which are the most relevant safety issues of psychotropic medications in people with COVID-19?

| **Population** | people with psychiatric conditions with or without comorbid medical conditions, particularly those associated with respiratory, cardiovascular, infective, immune, haematological, haemostatic, or neuro-psychiatric manifestations comparable to those observed in people with COVID-19. |
| --- | --- |
| **Interventions/exposure** | all antidepressants, antipsychotics, anxiolytics, and selected antiepileptics used for the treatment of mood disorders (<https://www.whocc.no/atc_ddd_index/>) and all medical treatments employed on- or off-label for COVID-19, as routinely used in current clinical practice protocols and currently undergoing rigorous experimental protocol, as reported by the WHO landscape analysis of therapeutics for COVID-19 (<https://www.who.int/blueprint/priority-diseases/key-action/Table_of_therapeutics_Appendix_17022020.pdf?ua=1>). |
| **Comparisons** | placebo or treatment as usual or no exposure or head-to-head comparison between psychotropic medications. |
| **Outcomes** | respiratory risk (including acute respiratory distress syndrome); cardiovascular risk (including QTc prolongation, ischemic heart disease, sudden cardiac death, stroke); risk of immunity abnormalities (including risk of infections); risk of coagulation/haemostasis abnormalities (including venous thromboembolism or bleeding); risk of delirium. |

**Table S3. Search strategy**

updated to 6^th^ May 2020

| **Database** | **Synthax** | **Records retrieved** |
| --- | --- | --- |
| **PubMed** | (antipsychotic*[Title/Abstract] OR neuroleptic*[Title/Abstract] OR antidepressant*[Title/Abstract] OR antidepressive agent[Title/Abstract] OR benzodiazepine*[Title/Abstract] OR anxiolytic*[Title/Abstract] OR mood stabilizer*[Title/Abstract] OR antiepileptic[Title/Abstract] OR lithium[Title/Abstract]) AND (respiratory distress[Title/Abstract] OR ventilatory distress[Title/Abstract] OR respiratory impariment[Title/Abstract] OR ventilatory impairment[Title/Abstract] OR Lung Injury[Title/Abstract] OR ARDS[Title/Abstract] OR pneumonia[Title/Abstract] OR interstitial pneumonia[Title/Abstract] OR cariovascular[Title/Abstract] OR cardiac[Title/Abstract] OR acute heart disease[Title/Abstract] OR cardiac death[Title/Abstract] OR ischemic heart disease[Title/Abstract] OR coronary heart disease[Title/Abstract] OR myocardial infarction[Title/Abstract] OR QT*[Title/Abstract] OR QT-prolong*[Title/Abstract] OR Torsades de Pointes[Title/Abstract] OR Torsade de Pointes[Title/Abstract] OR arrhythm*[Title/Abstract] OR cerebrovascular[Title/Abstract] OR stroke[Title/Abstract] OR immun*[Title/Abstract] OR autoimmun*[Title/Abstract] OR systemic inflammation[Title/Abstract] OR infection*[Title/Abstract] OR infection risk[Title/Abstract] OR infective risk[Title/Abstract] OR coagul*[Title/Abstract] OR bleeding[Title/Abstract] OR hemorrag*[Title/Abstract] OR thrombo*[Title/Abstract] OR emboli*[Title/Abstract] OR blood[Title/Abstract] OR dyscrasia*[Title/Abstract] OR delirium[Title/Abstract] OR confusion*[Title/Abstract] OR behaviour*[Title/Abstract] OR agitat*[Title/Abstract]) Filters: Systematic Reviews, in the last 10 years | 607 |
| **Cochrane Library** | antipsychotic* OR neuroleptic* OR antidepressant* OR antidepressive agent OR benzodiazepine* OR anxiolytic* OR mood stabilizer* OR antiepileptic OR lithium in Title Abstract Keyword AND respiratory distress OR ventilatory distress OR respiratory impariment OR ventilatory impairment OR Lung Injury OR ARDS OR pneumonia OR interstitial pneumonia OR cariovascular OR cardiac OR acute heart disease OR cardiac death OR ischemic heart disease OR coronary heart disease OR myocardial infarction OR QT* OR QT-prolong* OR Torsades de Pointes OR Torsade de Pointes OR arrhythm* OR cerebrovascular OR stroke OR immun* OR autoimmun* OR systemic inflammation OR infection* OR infection risk OR infective risk OR coagul* OR bleeding OR hemorrag* OR thrombo* OR emboli* OR blood OR dyscrasia* OR delirium OR confusion* OR behaviour* OR agitat* in Title Abstract Keyword; Limited to the last 10 years | 267 |
| **Web of Science** | TI=(respiratory distress OR ventilatory distress OR respiratory impariment OR ventilatory impairment OR Lung Injury OR ARDS OR pneumonia OR interstitial pneumonia OR cariovascular OR cardiac OR acute heart disease OR cardiac death OR ischemic heart disease OR coronary heart disease OR myocardial infarction OR QT OR QTc OR QT-prolong* OR Torsades de Pointes OR Torsade de Pointes OR arrhythm* OR cerebrovascular OR stroke OR immun* OR autoimmun* OR systemic inflammation OR infection* OR infection risk OR infective risk OR coagul* OR bleeding OR hemorrag* OR thrombo* OR emboli* OR blood OR dyscrasia* OR delirium OR confusion* OR behaviour* OR agitat*) AND TI/TS=(antipsychotic* OR neuroleptic* OR antidepressant* OR antidepressive agent OR benzodiazepine* OR anxiolytic* OR mood stabilizer* OR antiepileptic OR lithium). Refined by: PUBLICATION YEARS: (2020 OR 2011 OR 2019 OR 2018 OR 2017 OR 2016 OR 2015 OR 2014 OR 2013 OR 2012) AND DOCUMENT TYPES: (REVIEW); Indexes=SCI-EXPANDED, SSCI, A&HCI, CPCI-S, CPCI-SSH, ESCI | 613 |

**Figure S1. PRISMA flow-chart**


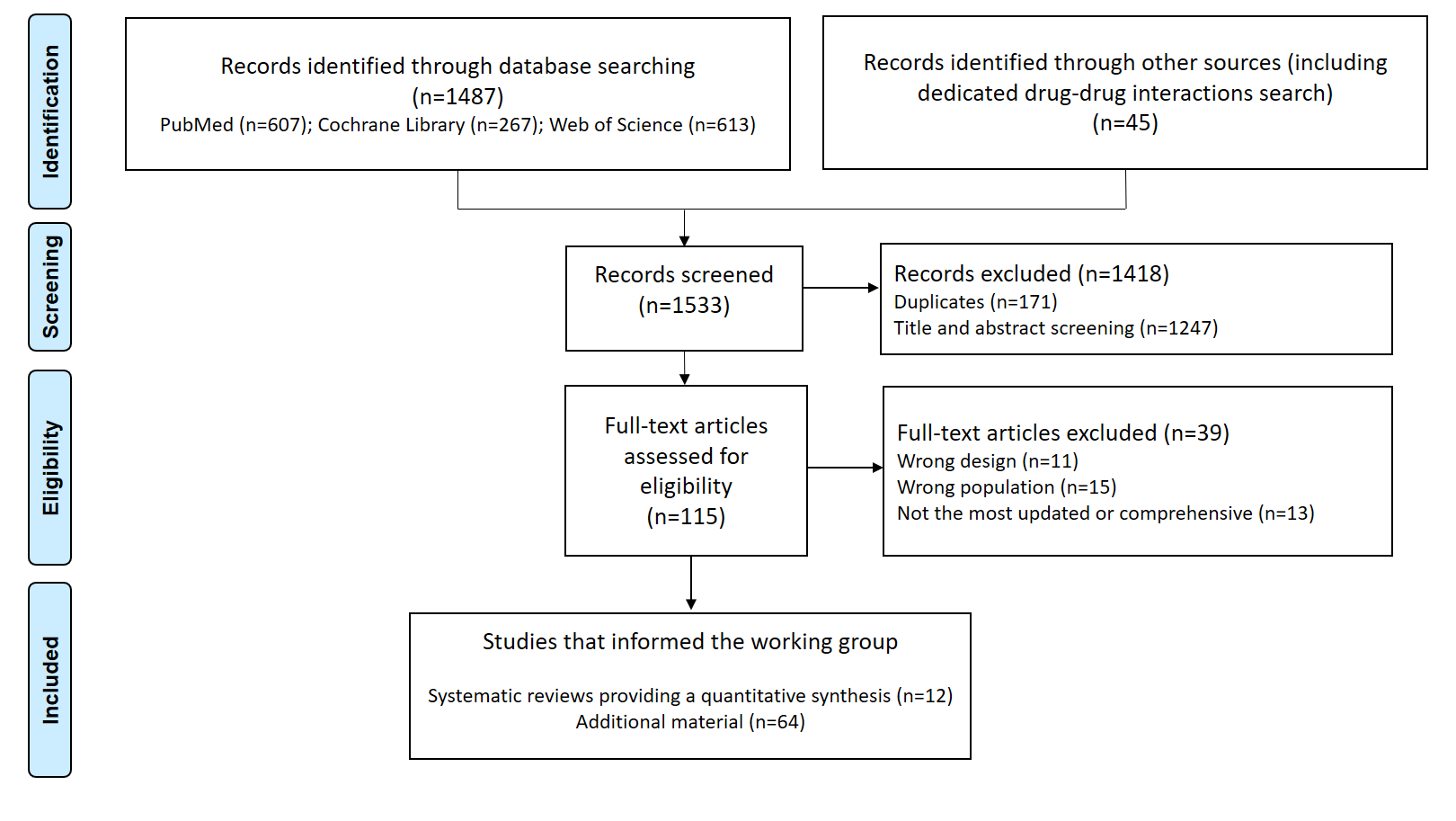


**Table S4. List of included studies**

| **Type** | **N. of articles included** | **List** |
| --- | --- | --- |
| Systematic reviews providing a quantitative synthesis | 12 | 1. Clegg A, Young JB. Which medications to avoid in people at risk of delirium: a systematic review. Age Ageing. 2011;40(1):23‐29. doi:10.1093/ageing/afq140 2. Dragioti E, Solmi M, Favaro A, et al. Association of Antidepressant Use With Adverse Health Outcomes: A Systematic Umbrella Review [published online ahead of print, 2019 Oct 2]. *JAMA Psychiatry*. 2019;76(12):1241‐1255. doi:10.1001/jamapsychiatry.2019.2859 3. Dzahini O, Singh N, Taylor D, Haddad PM. Antipsychotic drug use and pneumonia: Systematic review and meta-analysis. *J Psychopharmacol*. 2018;32(11):1167‐1181. doi:10.1177/0269881118795333 4. Huhn M, Nikolakopoulou A, Schneider-Thoma J, et al. Comparative efficacy and tolerability of 32 oral antipsychotics for the acute treatment of adults with multi-episode schizophrenia: a systematic review and network meta-analysis [published correction appears in Lancet. 2019 Sep 14;394(10202):918]. *Lancet*. 2019;394(10202):939‐951. doi:10.1016/S0140-6736(19)31135-3 5. Kunutsor SK, Seidu S, Khunti K. Depression, antidepressant use, and risk of venous thromboembolism: systematic review and meta-analysis of published observational evidence. *Ann Med*. 2018;50(6):529‐537. doi:10.1080/07853890.2018.1500703 6. Lu XM, Zhu JP, Zhou XM. The effect of benzodiazepines on insomnia in patients with chronic obstructive pulmonary disease: a meta-analysis of treatment efficacy and safety. *Int J Chron Obstruct Pulmon Dis*. 2016;11:675‐685. Published 2016 Apr 4. doi:10.2147/COPD.S98082 7. Ostuzzi G, Turrini G, Gastaldon C, et al. Efficacy and acceptability of antidepressants in patients with ischemic heart disease: systematic review and meta-analysis. *Int Clin Psychopharmacol*. 2019;34(2):65‐75. doi:10.1097/YIC.0000000000000248 8. Papola D, Ostuzzi G, Gastaldon C, et al. Antipsychotic use and risk of life-threatening medical events: umbrella review of observational studies. *Acta Psychiatr Scand*. 2019;140(3):227‐243. doi:10.1111/acps.13066 9. Pollok J, van Agteren JE, Esterman AJ, Carson-Chahhoud KV. Psychological therapies for the treatment of depression in chronic obstructive pulmonary disease. *Cochrane Database Syst Rev*. 2019;3(3):CD012347. Published 2019 Mar 6. doi:10.1002/14651858.CD012347.pub2 10. Schneider-Thoma J, Efthimiou O, Bighelli I, et al. Second-generation antipsychotic drugs and short-term somatic serious adverse events: a systematic review and meta-analysis. *Lancet Psychiatry*. 2019;6(9):753‐765. doi:10.1016/S2215-0366(19)30223-8 11. Sun GQ, Zhang L, Zhang LN, Wu Z, Hu DF. Benzodiazepines or related drugs and risk of pneumonia: A systematic review and meta-analysis. *Int J Geriatr Psychiatry*. 2019;34(4):513‐521. doi:10.1002/gps.5048 12. Wu YC, Tseng PT, Tu YK, et al. Association of Delirium Response and Safety of Pharmacological Interventions for the Management and Prevention of Delirium: A Network Meta-analysis. *JAMA Psychiatry*. 2019;76(5):526‐535. doi:10.1001/jamapsychiatry.2018.4365 |

**Table S5. Additional material that informed the working group**

| **Outcome of interest** | **N. of articles included** | **List** |
| --- | --- | --- |
| Respiratory risk | 13 | 1. Bajwah S, Davies JM, Tanash H, Currow DC, Oluyase AO, Ekström M. Safety of benzodiazepines and opioids in interstitial lung disease: a national prospective study. *Eur Respir J*. 2018;52(6):1801278. Published 2018 Dec 6. doi:10.1183/13993003.01278-2018 2. Galling B, Roldán A, Hagi K, et al. Antipsychotic augmentation vs. monotherapy in schizophrenia: systematic review, meta-analysis and meta-regression analysis. *World Psychiatry*. 2017;16(1):77–89. doi:10.1002/wps.20387 3. Griffin CE 3rd, Kaye AM, Bueno FR, Kaye AD. Benzodiazepine pharmacology and central nervous system-mediated effects. *Ochsner J*. 2013;13(2):214–223. 4. Kang M, Galuska MA, Ghassemzadeh S. Benzodiazepine Toxicity [Updated 2020 Apr 11]. In: StatPearls [Internet]. Treasure Island (FL): StatPearls Publishing; 2020. 5. Kaplan J, Schwartz AC, Ward MC. Clozapine-Associated Aspiration Pneumonia: Case Series and Review of the Literature. Psychosomatics 2018;59:199-203. 6. Kirmeier E, Eriksson LI, Lewald H, et al. Post-anaesthesia pulmonary complications after use of muscle relaxants (POPULAR): a multicentre, prospective observational study [published correction appears in Lancet Respir Med. 2018 Nov 6]. *Lancet Respir Med*. 2019;7(2):129–140. doi:10.1016/S2213-2600(18)30294-7 7. NICE 2009. Depression in adults: recognition and management. Clinical guideline [CG90] Publisher: National Institute for Health and Care Excellence URL: https://www.nice.org.uk/guidance/CG90 8. Shah FA, Girard TD, Yende S. Limiting sedation for patients with acute respiratory distress syndrome - time to wake up. Current Opinion in Critical Care 2017;23:45-51. 9. Torbic H, Duggal A. Antipsychotics, Delirium, and Acute Respiratory Distress Syndrome: What Is the Link? *Pharmacotherapy*. 2018;38(4):462‐469. doi:10.1002/phar.2093 10. Vozoris NT, Fischer HD, Wang X, et al. Benzodiazepine drug use and adverse respiratory outcomes among older adults with COPD. *Eur Respir J*. 2014;44(2):332–340. doi:10.1183/09031936.00008014 11. Vozoris NT, Wang X, Austin PC, et al. Serotonergic antidepressant use and morbidity and mortality among older adults with COPD. *Eur Respir J*. 2018;52(1):1800475. Published 2018 Jul 27. doi:10.1183/13993003.00475-2018 12. Wang MT, Tsai CL, Lin CW, Yeh CB, Wang YH, Lin HL. Association Between Antipsychotic Agents and Risk of Acute Respiratory Failure in Patients With Chronic Obstructive Pulmonary Disease. *JAMA Psychiatry*. 2017;74(3):252–260. doi:10.1001/jamapsychiatry.2016.3793 13. Yohannes AM, Alexopoulos GS. Pharmacological treatment of depression in older patients with chronic obstructive pulmonary disease: impact on the course of the disease and health outcomes. *Drugs Aging*. 2014;31(7):483‐492. doi:10.1007/s40266-014-0186-0 |
| Cardiovascular risk | 12 | 1. Balon R, Rafanelli C, Sonino N. Benzodiazepines: A Valuable Tool in the Management of Cardiovascular Conditions. *Psychother Psychosom*. 2018;87(6):327–330. doi:10.1159/000493015 2. Barbui C, Bighelli I, Carrà G, et al. Antipsychotic Dose Mediates the Association between Polypharmacy and Corrected QT Interval. *PLoS One*. 2016;11(2):e0148212. Published 2016 Feb 3. doi:10.1371/journal.pone.0148212 3. Beach SR, Celano CM, Sugrue AM, et al. QT Prolongation, Torsades de Pointes, and Psychotropic Medications: A 5-Year Update. *Psychosomatics*. 2018;59(2):105‐122. doi:10.1016/j.psym.2017.10.009 4. Bindraban AN, Rolvink J, Berger FA, et al. Development of a risk model for predicting QTc interval prolongation in patients using QTc-prolonging drugs. *Int J Clin Pharm*. 2018;40(5):1372–1379. doi:10.1007/s11096-018-0692-y 5. Carvalho AF, Sharma MS, Brunoni AR, Vieta E, Fava GA. The Safety, Tolerability and Risks Associated with the Use of Newer Generation Antidepressant Drugs: A Critical Review of the Literature. *Psychother Psychosom*. 2016;85(5):270‐288. doi:10.1159/000447034 6. Correll CU, Detraux J, De Lepeleire J, De Hert M. Effects of antipsychotics, antidepressants and mood stabilizers on risk for physical diseases in people with schizophrenia, depression and bipolar disorder. *World Psychiatry*. 2015;14(2):119‐136. doi:10.1002/wps.20204 7. FDA. FDA-Approved Drugs. US Food and Drug Admninistration; 2020. <https://www.accessdata.fda.gov/scripts/cder/daf/> 8. Hasnain M, Vieweg WV. QTc interval prolongation and torsade de pointes associated with second-generation antipsychotics and antidepressants: a comprehensive review. *CNS Drugs*. 2014;28(10):887‐920. doi:10.1007/s40263-014-0196-9 9. Mehta N, Vannozzi R. Lithium-induced electrocardiographic changes: A complete review. Clinical Cardiology 2017;40:1363-7. 10. Polcwiartek C, Sneider B, Graff C, et al. The cardiac safety of aripiprazole treatment in patients at high risk for torsade: a systematic review with a meta-analytic approach. Psychopharmacology 2015;232:3297-308. 11. Schwartz PJ, Woosley RL. Predicting the Unpredictable: Drug-Induced QT Prolongation and Torsades de Pointes. *J Am Coll Cardiol*. 2016;67(13):1639–1650. doi:10.1016/j.jacc.2015.12.063 12. Takeuchi H, Suzuki T, Remington G, Uchida H. Antipsychotic Polypharmacy and Corrected QT Interval: A Systematic Review. Canadian journal of psychiatry Revue canadienne de psychiatrie 2015;60:215-22. |
| Risk of infections | 20 | 1. Brandt J, Leong C. Benzodiazepines and Z-Drugs: An Updated Review of Major Adverse Outcomes Reported on in Epidemiologic Research. *Drugs R D*. 2017;17(4):493‐507. doi:10.1007/s40268-017-0207-7 2. Curtis BR. Drug-induced immune neutropenia/agranulocytosis. *Immunohematology*. 2014;30(2):95–101. 3. da Rosa TF, Machado CS, Serafin MB, et al. Repositioning or Redirection of Antidepressant Drugs in the Treatment of Bacterial and Fungal Infections [published online ahead of print, 2019 May 8]. *Am J Ther*. 2019;10.1097/MJT.0000000000001001. doi:10.1097/MJT.0000000000001001 4. de Leon J, Sanz EJ, Norén GN, De Las Cuevas C. Pneumonia may be more frequent and have more fatal outcomes with clozapine than with other second-generation antipsychotics. *World Psychiatry*. 2020;19(1):120–121. doi:10.1002/wps.20707 5. Deidda A, Pisanu C, Micheletto L, Bocchetta A, Del Zompo M, Stochino ME. Interstitial lung disease induced by fluoxetine: Systematic review of literature and analysis of Vigiaccess, Eudravigilance and a national pharmacovigilance database. Pharmacological Research 2017;120:294-301. 6. Gałecki P, Mossakowska-Wójcik J, Talarowska M. The anti-inflammatory mechanism of antidepressants - SSRIs, SNRIs. *Prog Neuropsychopharmacol Biol Psychiatry*. 2018;80(Pt C):291–294. doi:10.1016/j.pnpbp.2017.03.016 7. Howland RH. More on antidepressant drugs and infectious disease. *J Psychosoc Nurs Ment Health Serv*. 2013;51(10):11–13. doi:10.3928/02793695-20130903-02 8. Köhler CA, Freitas TH, Stubbs B, et al. Peripheral Alterations in Cytokine and Chemokine Levels After Antidepressant Drug Treatment for Major Depressive Disorder: Systematic Review and Meta-Analysis. *Mol Neurobiol*. 2018;55(5):4195–4206. doi:10.1007/s12035-017-0632-1 9. Levin GM, DeVane CL. A review of cyclic antidepressant-induced blood dyscrasias. *Ann Pharmacother*. 1992;26(3):378‐383. doi:10.1177/106002809202600313 10. Murru A, Popovic D, Pacchiarotti I, Hidalgo D, León-Caballero J, Vieta E. Management of adverse effects of mood stabilizers. *Curr Psychiatry Rep*. 2015;17(8):603. doi:10.1007/s11920-015-0603-z 11. Nooijen PM, Carvalho F, Flanagan RJ. Haematological toxicity of clozapine and some other drugs used in psychiatry. *Hum Psychopharmacol*. 2011;26(2):112–119. doi:10.1002/hup.1181 12. Ponsford M, Castle D, Tahir T, et al. Clozapine is associated with secondary antibody deficiency [published online ahead of print, 2018 Sep 27]. *Br J Psychiatry*. 2018;214(2):1–7. doi:10.1192/bjp.2018.152 13. Ponsford M, Castle D, Tahir T, et al. Clozapine is associated with secondary antibody deficiency. The British journal of psychiatry : the journal of mental science 2018;214:1-7. 14. Rochester MP, Kane AM, Linnebur SA, Fixen DR. Evaluating the risk of QTc prolongation associated with antidepressant use in older adults: a review of the evidence. Therapeutic Advances in Drug Safety 2018;9:297-308. 15. Romeo B, Brunet-Lecomte M, Martelli C, Benyamina A. Kinetics of Cytokine Levels during Antipsychotic Treatment in Schizophrenia: A Meta-Analysis. *Int J Neuropsychopharmacol*. 2018;21(9):828–836. doi:10.1093/ijnp/pyy062 16. Stapel B, Sieve I, Falk CS, Bleich S, Hilfiker-Kleiner D, Kahl KG. Second generation atypical antipsychotics olanzapine and aripiprazole reduce expression and secretion of inflammatory cytokines in human immune cells. *J Psychiatr Res*. 2018;105:95–102. doi:10.1016/j.jpsychires.2018.08.017 17. Stapel B, Sieve I, Falk CS, Bleich S, Hilfiker-Kleiner D, Kahl KG. Second generation atypical antipsychotics olanzapine and aripiprazole reduce expression and secretion of inflammatory cytokines in human immune cells. Journal of psychiatric research 2018;105:95-102. 18. Sultana J, Calabro M, Garcia-Serna R, et al. Biological substantiation of antipsychotic-associated pneumonia: Systematic literature review and computational analyses. Plos One 2017;12 19. Szałach ŁP, Lisowska KA, Cubała WJ. The Influence of Antidepressants on the Immune System. *Arch Immunol Ther Exp (Warsz)*. 2019;67(3):143–151. doi:10.1007/s00005-019-00543-8 20. Wiciński M, Węclewicz MM. Clozapine-induced agranulocytosis/granulocytopenia: mechanisms and monitoring. *Curr Opin Hematol*. 2018;25(1):22–28. doi:10.1097/MOH.0000000000000391 |
| Coagulation risk | 3 | 1. Andrade C, Sandarsh S, Chethan KB, Nagesh KS. Serotonin reuptake inhibitor antidepressants and abnormal bleeding: a review for clinicians and a reconsideration of mechanisms. *J Clin Psychiatry*. 2010;71(12):1565‐1575. doi:10.4088/JCP.09r05786blu 2. Halperin D, Reber G. Influence of antidepressants on hemostasis. *Dialogues Clin Neurosci*. 2007;9(1):47–59. 3. Lal LS, Zhuang A, Hung F, Feng C, Arbuckle R, Fisch MJ. Evaluation of drug interactions in patients treated with antidepressants at a tertiary care cancer center. *Support Care Cancer*. 2012;20(5):983‐989. doi:10.1007/s00520-011-1170-4 |
| Risk of delirium | 2 | 1. Alagiakrishnan K, Wiens CA. An approach to drug induced delirium in the elderly. *Postgrad Med J*. 2004;80(945):388–393. doi:10.1136/pgmj.2003.017236 2. Hui D. Benzodiazepines for agitation in patients with delirium: selecting the right patient, right time, and right indication. Current Opinion in Supportive and Palliative Care 2018;12:489-94. |
| Drug-drug interactions | 14 | 1. Belmonte C, Ochoa D, Román M, et al. Evaluation of the Relationship Between Pharmacokinetics and the Safety of Aripiprazole and Its Cardiovascular Effects in Healthy Volunteers. Journal of clinical psychopharmacology 2016;36:608-14. 2. Dickmann LJ, Patel SK, Rock DA, Wienkers LC, Slatter JG. Effects of interleukin-6 (IL-6) and an anti-IL-6 monoclonal antibody on drug-metabolizing enzymes in human hepatocyte culture. Drug metabolism and disposition: the biological fate of chemicals 2011;39:1415-22. 3. Faber MS, Fuhr U. Time response of cytochrome P450 1A2 activity on cessation of heavy smoking. Clinical pharmacology and therapeutics 2004;76:178-84. 4. Finley PR. Drug Interactions with Lithium: An Update. Clinical pharmacokinetics 2016;55:925-41. 5. Hiemke C, Bergemann N, Clement HW, et al. Consensus Guidelines for Therapeutic Drug Monitoring in Neuropsychopharmacology: Update 2017. Pharmacopsychiatry 2018;51:e1. 6. Islam M, Frye RF, Richards TJ, et al. Differential effect of IFNalpha-2b on the cytochrome P450 enzyme system: a potential basis of IFN toxicity and its modulation by other drugs. Clinical cancer research: an official journal of the American Association for Cancer Research 2002;8:2480-7. 7. Molanaei H, Qureshi AR, Heimbürger O, et al. Inflammation down-regulates CYP3A4-catalysed drug metabolism in hemodialysis patients. BMC Pharmacology and Toxicology 2018;19:33. 8. Roncero C, Villegas JL, Martinez-Rebollar M, Buti M. The pharmacological interactions between direct-acting antivirals for the treatment of chronic hepatitis c and psychotropic drugs. Expert Review of Clinical Pharmacology 2018;11:999-1030. 9. Schoretsanitis G, Spina E, Hiemke C, de Leon J. A systematic review and combined analysis of therapeutic drug monitoring studies for oral paliperidone. Expert Review of Clinical Pharmacology 2018;11:625-39. 10. Shafiekhani M, Mirjalili M, Vazin A. Psychotropic drug therapy in patients in the intensive care unit - usage, adverse effects, and drug interactions: a review. Therapeutics and Clinical Risk Management 2018;14:1799-812. 11. Siccardi M, Marzolini C, Seden K, et al. Prediction of drug-drug interactions between various antidepressants and efavirenz or boosted protease inhibitors using a physiologically based pharmacokinetic modelling approach. Clinical pharmacokinetics 2013;52:583-92. 12. Spina E, de Leon J. Potentially Clinically Relevant Pharmacodynamic Interactions Between Antiepileptic Drugs and Psychotropic Drugs: An Update. Current Pharmaceutical Design 2017;23:5625-38. 13. Spina E, Hiemke C, de Leon J. Assessing drug-drug interactions through therapeutic drug monitoring when administering oral second-generation antipsychotics. Expert Opinion on Drug Metabolism & Toxicology 2016;12:407-22. 14. Spina E, Pisani F, de Leon J. Clinically significant pharmacokinetic drug interactions of antiepileptic drugs with new antidepressants and new antipsychotics. Pharmacological Research 2016;106:72-86. |

**Table S6. List of excluded studies, with reason**

| **Reason for exclusion** | **N. of studies excluded** | **List** |
| --- | --- | --- |
| Wrong design | 11 | 1. Lavoie KL, Paine NJ, Pelletier R, et al. Relationship between antidepressant therapy and risk for cardiovascular events in patients with and without cardiovascular disease. *Health Psychol*. 2018;37(11):989‐999. doi:10.1037/hea0000602 2. Seligman F, Nemeroff CB. The interface of depression and cardiovascular disease: therapeutic implications. *Ann N Y Acad Sci*. 2015;1345:25‐35. doi:10.1111/nyas.12738 3. Kahl KG. Direct and indirect effects of psychopharmacological treatment on the cardiovascular system. *Horm Mol Biol Clin Investig*. 2018;36(1):/j/hmbci.2018.36.issue-1/hmbci-2018-0054/hmbci-2018-0054.xml. Published 2018 Nov 14. doi:10.1515/hmbci-2018-0054 4. Zhou C, Sui Y, Zhao W, et al. The critical interaction between valproate sodium and warfarin: case report and review. *BMC Pharmacol Toxicol*. 2018;19(1):60. Published 2018 Oct 1. doi:10.1186/s40360-018-0251-0 5. Herzig SJ, LaSalvia MT, Naidus E, et al. Antipsychotics and the Risk of Aspiration Pneumonia in Individuals Hospitalized for Nonpsychiatric Conditions: A Cohort Study. *J Am Geriatr Soc*. 2017;65(12):2580‐2586. doi:10.1111/jgs.15066 6. Dublin S, Walker RL, Jackson ML, et al. Use of opioids or benzodiazepines and risk of pneumonia in older adults: a population-based case-control study. *J Am Geriatr Soc*. 2011;59(10):1899‐1907. doi:10.1111/j.1532-5415.2011.03586.x 7. Park SI, An H, Kim A, Jang IJ, Yu KS, Chung JY. An analysis of QTc prolongation with atypical antipsychotic medications and selective serotonin reuptake inhibitors using a large ECG record database. Expert opinion on drug safety 2016;15:1013-9. 8. Ramalho D, Freitas J. Drug-induced life-threatening arrhythmias and sudden cardiac death: A clinical perspective of long QT, short QT and Brugada syndromes. Revista Portuguesa De Cardiologia 2018;37:435-46. 9. Dijkstra ME, van der Weiden CFS, Schol-Gelok S, et al. Venous thrombosis during olanzapine treatment: a complex association. *Neth J Med*. 2018;76(6):263‐268. 10. Vieweg WV, Hasnain M, Hancox JC, et al. Risperidone, QTc interval prolongation, and torsade de pointes: a systematic review of case reports. Psychopharmacology 2013;228:515-24. 11. Yagmur F, Ulusoy HB, Buyukoglan H, Kaya MG. Acute respiratory distress due to antipsychotic drugs. Pharmacopsychiatry 2010;43:118-9. |
| Wrong population | 15 | 1. Fraser GL, Devlin JW, Worby CP, et al. Benzodiazepine versus nonbenzodiazepine-based sedation for mechanically ventilated, critically ill adults: a systematic review and meta-analysis of randomized trials. *Crit Care Med*. 2013;41(9 Suppl 1):S30‐S38. doi:10.1097/CCM.0b013e3182a16898 2. Hill L, Lee KC. Pharmacotherapy considerations in patients with HIV and psychiatric disorders: focus on antidepressants and antipsychotics. *Ann Pharmacother*. 2013;47(1):75‐89. doi:10.1345/aph.1R343 3. Eshun-Wilson I, Siegfried N, Akena DH, Stein DJ, Obuku EA, Joska JA. Antidepressants for depression in adults with HIV infection. *Cochrane Database Syst Rev*. 2018;1(1):CD008525. Published 2018 Jan 22. doi:10.1002/14651858.CD008525.pub3 4. Yunusa I, Alsumali A, Garba AE, Regestein QR, Eguale T. Assessment of Reported Comparative Effectiveness and Safety of Atypical Antipsychotics in the Treatment of Behavioral and Psychological Symptoms of Dementia: A Network Meta-analysis. *JAMA Netw Open*. 2019;2(3):e190828. Published 2019 Mar 1. doi:10.1001/jamanetworkopen.2019.0828 5. Beller EM, van Driel ML, McGregor L, Truong S, Mitchell G. Palliative pharmacological sedation for terminally ill adults. Cochrane Database of Systematic Reviews 2015. 6. Conway A, Rolley J, Sutherland JR. Midazolam for sedation before procedures. Cochrane Database of Systematic Reviews 2016. 7. Creese B, Da Silva MV, Johar I, Ballard C. The modern role of antipsychotics for the treatment of agitation and psychosis in Alzheimer's disease. Expert review of neurotherapeutics 2018;18:461-7. 8. Finucane AM, Jones L, Leurent B, et al. Drug therapy for delirium in terminally ill adults. Cochrane Database of Systematic Reviews 2020. 9. Burry L, Mehta S, Perreault MM, et al. Antipsychotics for treatment of deliriumin hospitalised non-ICU patients. Cochrane Database of Systematic Reviews 2018. 10. Jutkowitz E, Brasure M, Fuchs E, et al. Care-Delivery Interventions to Manage Agitation and Aggression in Dementia Nursing Home and Assisted Living Residents: A Systematic Review and Meta-analysis. Journal of the American Geriatrics Society 2016;64:477-88. 11. Meagher DJ, McLoughlin L, Leonard M, Hannon N, Dunne C, O'Regan N. What do we really know about the treatment of delirium with antipsychotics? Ten key issues for delirium pharmacotherapy. *Am J Geriatr Psychiatry*. 2013;21(12):1223‐1238. doi:10.1016/j.jagp.2012.09.008 12. Al-Qadheeb NS, Balk EM, Fraser GL, et al. Randomized ICU trials do not demonstrate an association between interventions that reduce delirium duration and short-term mortality: a systematic review and meta-analysis. Critical care medicine 2014;42:1442-54. 13. Burry L, Hutton B, Williamson DR, et al. Pharmacological interventions for the treatment of delirium in critically ill adults. The Cochrane database of systematic reviews 2019;9:Cd011749. 14. Ostinelli EG, Brooke-Powney MJ, Li X, Adams CE. Haloperidol for psychosis-induced aggression or agitation (rapid tranquillisation). *Cochrane Database Syst Rev*. 2017;7(7):CD009377. Published 2017 Jul 31. doi:10.1002/14651858.CD009377.pub3 15. Ostinelli EG, Hussein M, Ahmed U, Rehman FU, Miramontes K, Adams CE. Risperidone for psychosis-induced aggression or agitation (rapid tranquillisation). *Cochrane Database Syst Rev*. 2018;4(4):CD009412. Published 2018 Apr 10. doi:10.1002/14651858.CD009412.pub2 |
| Not the most updated or comprehensive | 13 | 1. Hägg S, Spigset O. Antipsychotic-induced venous thromboembolism: a review of the evidence. *CNS Drugs*. 2002;16(11):765‐776. doi:10.2165/00023210-200216110-00005 2. Jackson JW, Schneeweiss S, VanderWeele TJ, Blacker D. Quantifying the role of adverse events in the mortality difference between first and second-generation antipsychotics in older adults: systematic review and meta-synthesis. *PLoS One*. 2014;9(8):e105376. Published 2014 Aug 20. doi:10.1371/journal.pone.0105376 3. Paciullo CA. Evaluating the association between clozapine and venous thromboembolism. *Am J Health Syst Pharm*. 2008;65(19):1825‐1829. doi:10.2146/ajhp070638 4. Neufeld KJ, Yue J, Robinson TN, Inouye SK, Needham DM. Antipsychotic Medication for Prevention and Treatment of Delirium in Hospitalized Adults: A Systematic Review and Meta-Analysis [published correction appears in J Am Geriatr Soc. 2016 Oct;64(10):2171-2173]. *J Am Geriatr Soc*. 2016;64(4):705‐714. doi:10.1111/jgs.14076 5. Acharya S, Bussel JB. Hematologic toxicity of sodium valproate. *J Pediatr Hematol Oncol*. 2000;22(1):62‐65. doi:10.1097/00043426-200001000-00012 6. Nosè M, Recla E, Trifirò G, Barbui C. Antipsychotic drug exposure and risk of pneumonia: a systematic review and meta-analysis of observational studies. *Pharmacoepidemiol Drug Saf*. 2015;24(8):812‐820. doi:10.1002/pds.3804 7. Devlin JW, Mallow-Corbett S, Riker RR. Adverse drug events associated with the use of analgesics, sedatives, and antipsychotics in the intensive care unit. *Crit Care Med*. 2010;38(6 Suppl):S231‐S243. doi:10.1097/CCM.0b013e3181de125a 8. Trifirò G, Spina E, Gambassi G. Use of antipsychotics in elderly patients with dementia: do atypical and conventional agents have a similar safety profile? *Pharmacol Res*. 2009;59(1):1‐12. doi:10.1016/j.phrs.2008.09.017 9. Guina J, Merrill B. Benzodiazepines I: Upping the Care on Downers: The Evidence of Risks, Benefits and Alternatives. *J Clin Med*. 2018;7(2):17. Published 2018 Jan 30. doi:10.3390/jcm7020017 10. Griffin CE, 3rd, Kaye AM, Bueno FR, Kaye AD. Benzodiazepine pharmacology and central nervous system-mediated effects. The Ochsner journal 2013;13:214-23. 11. Kogut C, Crouse EB, Vieweg WV, et al. Selective serotonin reuptake inhibitors and torsade de pointes: new concepts and new directions derived from a systematic review of case reports. Therapeutic advances in drug safety 2013;4:189-98. 12. Liapikou A, Cilloniz C, Torres A. Drugs that increase the risk of community-acquired pneumonia: a narrative review. Expert opinion on drug safety 2018;17:991-1003. 13. Maslej MM, Bolker BM, Russell MJ, et al. The Mortality and Myocardial Effects of Antidepressants Are Moderated by Preexisting Cardiovascular Disease: A Meta-Analysis. *Psychother Psychosom*. 2017;86(5):268‐282. doi:10.1159/000477940 |

**Table S7. AMSTAR-2 of included systematic reviews**

The quality of included SRs was assessed using AMSTAR-2 (A Measurement Tool to Assess Systematic Reviews), a 16-point assessment tool of the methodological quality of SRs. AMSTAR-2 assesses systematic reviews on the following categories: (1) formulation of the research question; (2) a priori design provided; (3) explanation for the chosen study design of the included studies; (4) comprehensive literature search; (5) study selection; (6) data extraction; (7) presence of a list of excluded studies, along with reason for exclusion; (8) comprehensive description of the main features of the included studies; (9) risk of bias assessment; (10) information about the sources of funding for the studies included in the review; (11) methods for statistical combination of results; (12) assessment of the potential impact of risk of bias of individual studies on the meta-analysis result; (13) discussion/interpretation of the potential impact of risk of bias of individual studies on the meta-analysis result; (14) discussion of the heterogeneity observed in the study results; (15) likelihood of publication bias; and (16) declaration of study authors’ conflict of interest. Of these 16 domains, seven can particularly affect the validity of the review and its conclusion and are considered ‘critical domains’ (domains 2–4–7–9–11–13–15). Each item allows for the following response options: yes, partial yes or no. AMSTAR 2 is not intended to be scored. AMSTAR-2 proposes a scheme for interpreting weaknesses detected in critical and non-critical items:

- ‘high-quality’ studies show no or one noncritical weakness;
- ‘moderate-quality’ studies show more than one non-critical weakness but no critical flaws;
- ‘low-quality’ studies show one critical flaw with or without non-critical weaknesses;
- ‘critically low’ quality studies show more than one critical flaw with or without non-critical weaknesses.

| Systematic reviews | AMSTAR-2 Domain | | | | | | | | | | | | | | | | Reporting quality of each systematic review |
| --- | --- | --- | --- | --- | --- | --- | --- | --- | --- | --- | --- | --- | --- | --- | --- | --- | --- |
|  | 1 | 2 | 3 | 4 | 5 | 6 | 7 | 8 | 9 | 10 | 11 | 12 | 13 | 14 | 15 | 16 |  |
| Clegg et al. 2011 |  |  |  |  |  |  |  |  |  |  | NA | NA | NA | NA | NA |  | Critically low |
| Dzahini et al. 2018 |  |  |  |  |  |  |  |  |  |  |  |  |  |  |  |  | Critically low |
| Dragioti et al. 2019 |  |  |  |  |  |  |  |  |  |  |  |  |  |  |  |  | Coronary disease with TCA*:  Critically low |
|  |  |  |  |  |  |  |  |  |  |  |  |  |  |  |  |  | Acute heart disease with SSRI; cerebrovascular disease with TCA/SSRIꟸ:  Critically low |
|  |  |  |  |  |  |  |  |  |  |  |  |  |  |  |  |  | Myocardial infarction with AD‽:  Critically low |
|  |  |  |  |  |  |  |  |  |  |  |  |  |  |  |  |  | Coagulation risk with SSRIs and SNRIs†: Critically low |
| Huhn et al. 2019 |  |  |  |  |  |  |  |  |  |  |  |  |  |  |  |  | Low |
| Kunutson et al. 2018 |  |  |  |  |  |  |  |  |  |  |  |  |  |  |  |  | Critically low |
| Lu 2016 |  |  |  |  |  |  |  |  |  |  |  |  |  |  |  |  | Critically low |
| Ostuzzi et al. 2019 |  |  |  |  |  |  |  |  |  |  |  |  |  |  |  |  | High |
| Papola et al. 2019 |  |  |  |  |  |  |  |  |  |  |  |  |  |  |  |  | Cardiovascular outcome‡: Low |
|  |  |  |  |  |  |  |  |  |  |  |  |  |  |  |  |  | Coagulation outcome∫: Low |
| Pollok et al. 2018 |  |  |  |  |  |  |  |  |  |  |  |  |  |  |  |  | High |
| Schneider-Thoma et al. 2019 |  |  |  |  |  |  |  |  |  |  |  |  |  |  |  |  | Low |
| Sun et al. 2018 |  |  |  |  |  |  |  |  |  |  |  |  |  |  |  |  | Critically low |
| Wu et al. 2019 |  |  |  |  |  |  |  |  |  |  |  |  |  |  |  |  | Low |

|  | Criterion met |
| --- | --- |
|  | Criterion not met |
|  | Criterion partially met |
| NA | Not applicable |

‘Critical’ domains are: 4, 7, 9, 11, 13, 15.

* Oh SW, Kim J, Myung SK, Hwang SS, Yoon DH. Antidepressant use and risk of coronary heart disease: meta-analysis of observational studies. Br J Clin Pharmacol. 2014;78(4):727-737

ꟸ Biffi A, Scotti L, Corrao G. Use of antidepressants and the risk of cardiovascular and cerebrovascular disease: a meta-analysis of observational studies. Eur J Clin Pharmacol. 2017;73(4):487-497.

‽ Undela K, Parthasarathi G, John SS. Impact of antidepressants use on risk of myocardial infarction: a systematic review and meta-analysis. Indian J Pharmacol. 2015;47(3):256-262.

†Laporte S, Chapelle C, Caillet P, et al. Bleeding risk under selective serotonin reuptake inhibitor (SSRI) antidepressants: ameta-analysis of observational studies. Pharmacol Res. 2017;118: 19-32.

‡Hsu WT, Esmaily-Fard A, Lai CC et al. Antipsychotics and the risk of cerebrovascular accident: a systematic review and meta-analysis of observational studies. J Am Med Dir Assoc 2017;18:692–699.

∫ Barbui C, Conti V, Cipriani A. Antipsychotic drug exposure and risk of venous thromboembolism: a systematic review and meta-analysis of observational studies. Drug Saf 2014;37:79–90.

| **Table S8. Drug-drug interactions table** | | | | | | | | | | | | | | | |
| --- | --- | --- | --- | --- | --- | --- | --- | --- | --- | --- | --- | --- | --- | --- | --- |
|  |  | **Antivirals** | | | | | **Antimalarial drugs^1^** | | **Antibiotics** | | | **Antirheumatics** | | **Other** | |
|  | Lopinavir/  Ritonavir^3^ | Ribavirin | Darunavir/  Cobicistat | Emtricitabine/  Tenofovir | Remdesivir | Ruxolitinib (RX) | Chloroquine | H-Chloroquine | Sulfamethoxazole/  trimethoprim | Azithromycin | Clarithromycin | Tocilizumab | Baricitinib | IFN α-2b | Heparin |
|  | **ANTIDEPRESSANTS** | | | | | | | | | | |  | |  | |
| Amitriptyline | QTc, ↑Amitriptyline, sedation |  | ↑Amitriptyline |  | ↑Amitriptyline |  | QTc ↑Amitriptyline | QTc | QTc, **⊗** AD effects | QTc | QTc, ↑Amitriptyline |  |  |  |  |
| Bupropion | ↓Bupropion, SS |  | ↑Cobicistat, ↑Darunavir |  |  |  | ↑Chloroquine | ↑H-Chloroquine |  |  |  |  |  |  |  |
| Citalopram | QTc, ↓Citalopram |  | ↑Citalopram |  | ↑Citalopram |  | QTc | QTc | QTc | QTc | QTc |  |  |  | Bleeding |
| Clomipramine | QTc, ↕Clomipramine |  | ↑Clomipramine |  | ↑Clomipramine |  | QTc, ↑Clomipramine | QTc | QTc, **⊗** AD effects | QTc | QTc, ↑Clomipramine |  |  |  |  |
| Duloxetine | ↕ Duloxetine |  | ↑Duloxetine |  | ↑Duloxetine |  | ↑Duloxetine |  |  |  |  | ↑ Duloxetine |  | adverse drug reactions | Bleeding |
| Escitalopram | QTc, ↓Escitalopram, SS |  | ↑Escitalopram, ↑Cobicistat |  | ↑Escitalopram |  | QTc | QTc | QTc | QTc | QTc |  |  |  | Bleeding |
| Fluoxetine | ↑Ritonavir, ↑Fluoxetine, SS |  | ↑Cobicistat, ↑Fluoxetine |  | ↑ | ↑  RX | ↑Fluoxetine |  | ↑Fluoxetine | QTc | ↑Fluoxetine, Delirium |  |  |  | Bleeding |
| Fluvoxamine | ↕Fluvoxamine |  | ↑Fluvoxamine, ↑Darunavir, ↑Cobicistat |  | ↑Remdesivir  ↑Fluvoxamine |  | ↑Fluvoxamine |  | ↑Sulfamethoxazole |  | ↑Clarithromycin |  |  |  | Bleeding |
| Imipramine | QTc, ↕ Imipramine |  | ↑Imipramine |  | ↑ |  | QTc, ↑Imipramine | QTc | **⊗** AD effects | QTc | QTc, ↑Imipramine |  |  |  |  |
| Mirtazapine | ↑Mirtazapine |  | ↑Mirtazapine |  |  |  | ↑Mirtazapine |  |  |  | ↑Mirtazapine | ↑ liver enzymes |  |  |  |
| Nortriptyline | QTc, ↑Nortriptyline, |  | ↑Nortriptiline |  | ↑ Nortriptyline |  | QTc, ↑Nortriptyline | QTc | **⊗** AD effects | QTc |  |  |  |  |  |
| Paroxetine | QTc,  ↑Paroxetine |  | ↕ Paroxetine, ↑Cobicistat |  | ↑ Paroxetine |  | Qtc, ↑Paroxetine | QTc |  | QTc | ↑Paroxetine  QTc |  |  |  | Bleeding |
| Sertraline | ↕ Sertraline |  | ↕ Sertraline |  | ↑Sertraline |  |  |  |  |  | ↑Sertraline |  |  |  | Bleeding |
| Trazodone | ↑Trazodone, SS, Nausea, ↓BP, Syncope |  | ↑Trazodone |  |  |  | ↑Trazodone |  |  | QTc | QTc, ↑Trazodone |  |  |  |  |
| Venlafaxine | QTc, ↑Venlafaxine |  | ↑ Venlafaxine |  | ↑Venlafaxine |  | QTc | QTc | QTc | QTc | QTc, ↑Venlafaxine |  |  |  | Bleeding |
| Vortioxetine | ↑Vortioxetine |  | ↑Vortioxetine |  | ↑ Vortioxetine |  | ↑Vortioxetine |  |  |  | ↑Vortioxetine |  |  |  |  |
|  | **ANTIPSYCHOTICS** | | | | | | | | | | |  | |  | |
| Aripiprazole | ↑Aripiprazole |  | ↑Aripiprazole |  | ↑ Aripiprazole |  | ↑Aripiprazole |  |  |  | ↑Aripiprazole |  |  |  |  |
| Asenapine | ↕Asenapine |  | ↑Asenapine |  | ↑ Asenapine |  |  |  |  |  |  |  |  |  |  |
| Brexpiprazole | ↑Brexpiprazole |  | ↑Brexpiprazole |  | ↑ Brexiprazole |  | ↑Brexpiprazole |  |  |  |  |  |  |  |  |
| Cariprazine | QTc, ↑Cariprazine |  | ↑Cariprazine |  |  |  | ↑Cariprazine |  |  |  | QTc, ↑Cariprazine |  |  |  |  |
| Chlorpromazine | QTc, ↑Chlorpromazine |  |  |  |  |  | QTc, ↑Chlorpromazine | QTc | QTc | QTc | QTc |  |  |  |  |
| Clotiapine |  |  |  |  |  |  |  |  |  |  |  |  |  |  |  |
| Clozapine | QTc, ↓Clozapine |  | ↑Clozapine |  | ↑ Clozapine |  | QTc, Agranulocytosis | QTc, Agranulocytosis | QTc, Agranulocytosis | QTc, Agranulocytosis | QTc, ↑Clozapine Seizures |  |  |  |  |
| Haloperidol | QTc, ↑Haloperidol |  | ↑Haloperidol |  | ↑ Haloperidol |  | QTc, ↑Haloperidol | QTc | QTc | QTc | QTc, ↑Haloperidol |  |  |  |  |
| Lurasidone | **[C]**  ↑Lurasidone |  | **[C]**  ↑Lurasidone, CA |  |  |  |  |  |  |  | ↑Lurasidone |  |  |  |  |
| Olanzapine | QTc, ↓Olanzapine, Met/SE, ↑Liver enzymes, **⊗** AP effects |  | Met/SE |  | ↑ Olanzapine |  | QTc | QTc | QTc, Neutropenia Pancreatitis, Falls ↑Liver enzymes | QTc, ↑Liver enzymes | QTc, ↑Liver enzymes |  |  |  |  |
| Paliperidone |  |  |  |  |  |  |  |  |  |  |  |  |  |  |  |
| Pimozide | **[C]**  QTc, ↑Pimozide |  | **[C]**  ↑Pimozide, CA |  | ↑ |  | QTc  ↑Pimozide | QTc | QTc | QTc | **[C]**  QTc, ↑Pimozide |  |  |  |  |
| Promazine | ↕Promazine |  | ↑Promazine |  |  |  | QTc, ↑Promazine | QTc | QTc | QTc | QTc, ↑Promazine |  |  |  |  |
| Quetiapine | QTc, ↑Quetiapine, +Sedation, Med/SE |  | **[C]**  ↑Quetiapine |  |  |  | QTc | QTc | QTc | QTc | QTc, ↑Quetiapine |  |  |  |  |
| Risperidone | QTc, ↑Risperidone, (toxicity) |  | ↑Risperidone, ↑Liver enzymes |  | ↑ Risperidone |  | QTc, ↑Risperidone | QTc | QTc | QTc | QTc |  |  |  |  |
| Tiapride | QTc |  |  |  |  |  | QTc | QTc | QTc |  |  |  |  |  |  |
|  | **MOOD STABILIZERS** | | | | | | | | | | |  | |  | |
| Carbamazepine | ↓Ritonavir, ↑Carbamazepine (toxicity) |  | ↓Cobicistat ↑Carbamazepine (toxicity) |  |  |  | ↓Chloroquine |  | ↑Carbamazepine, ↓Sulfamethoxazole, Hypersensitivty | ↓Azithromycin | ↑Carbamazepine (toxicity),  ↓Clarithromycin |  |  |  |  |
| Gabapentin |  |  |  |  |  |  |  |  |  |  |  |  |  |  |  |
| Lamotrigine | ↓Lamotrigine |  | Rash, GI |  |  |  |  |  |  |  |  |  |  |  |  |
| Lithium | QTc |  |  | NT |  |  | QTc | QTc | QTc, GI, NT |  |  |  |  |  |  |
| Pregabalin |  |  |  |  |  |  |  |  |  | Seizures | Rhabdomyolysis |  |  |  |  |
| Sodium Valproate | ↓Valproate,  ↑Ritonavir |  |  | GI |  |  |  |  | ↑Liver enzymes, GI | ↑Liver enzymes | ↑Liver enzymes |  |  |  |  |
|  | **BENZODIAZEPINES** | | | | | | | | | | |  | |  | |
| Alprazolam | ↑Alprazolam, RD+Sedation |  | ↑Alprazolam |  |  |  |  |  |  |  | ↑Alprazolam |  |  |  |  |
| Bromazepam | ↑Bromazepam, RD,  ↑Sedation |  | ↑Bromazepam |  | ↑ Bromazepam |  |  |  |  |  | ↑Bromazepam |  |  |  |  |
| Clonazepam | ↑Clonazepam |  | ↑Clonazepam |  |  |  |  |  |  |  | ↑Clonazepam |  |  |  |  |
| Delorazepam | ↑Delorazepam |  | ↑Delorazepam |  |  |  |  |  |  |  |  |  |  |  |  |
| Diazepam | ↑Diazepam |  | ↑Diazepam |  | ↑Diazepam |  | **⊗** Sedation |  |  |  | ↑Diazepam |  |  |  | ↑Diazepam |
| Etizolam | ↑Etizolam |  | ↑Etizolam |  |  |  |  |  |  |  |  |  |  |  |  |
| Flurazepam | ↑Flurazepam |  |  |  |  |  |  |  |  |  |  |  |  |  |  |
| Lorazepam | ↓Lorazepam |  |  |  |  |  |  |  |  |  |  |  |  |  |  |
| Lormetazepam | ↓Lormetazepam |  |  |  |  |  |  |  |  |  |  |  |  |  |  |
| Midazolam^4^ | **[C]**  ↑Midazolam, RD+Sedation |  | **[C]**  ↑Midazolam, RD+Sedation |  |  |  |  |  |  | ↑Midazolam | ↑Midazolam |  |  |  |  |
| Oxazepam | ↓Oxazepam |  |  |  |  |  |  |  |  |  |  |  |  |  |  |
| Triazolam | **[C]**  ↑Triazolam, RD |  | **[C]**  ↑Triazolam, RD+Sedation |  |  |  |  |  |  |  | ↑Triazolam |  |  |  |  |
| Zolpidem | ↑Zolpidem |  | ↑Zolpidem |  |  |  |  |  | ↑Zolpidem |  | ↑Zolpidem |  |  |  |  |

Abbreviations: AD: antidepressant effects; AP: antipsychotic effects; **[C]:** contraindicated combination according to the package insert; CA: cardiac arrhythmias; H-chloroquine: hydroxychloroquine; GI: gastrointestinal symptoms; Met/SE: metabolic side-effects; NT: nephrotoxicity phenomena; RX: Ruxolitinib; RD: respiratory depression; SS: serotonin syndrome.

Symbols: ↑: increased blood levels; ↓: decreased blood levels; ↕: As some of the antiviral products may have inducing as well as inhibiting properties on different CYP Isoenzymes, patterns of expected interactions may range from increased to decreased levels; ⊗: reduce effects; +: marked.

^1^ QT-prolongations by chloroquine and hydroxychloroquine have been shown to be restricted to high doses/concentrations.

^2^ When active metabolites were of clinical relevance, provided half-lives reflect both parent compound and active metabolite.

^3^ The interaction potential of ritonavir presents time- and dose-dependent patterns. Here we provide data on short-term administration of ritonavir low doses (100-400mg/day), which reflect clinical practices in treatment of COVID-19. When only evidence for high ritonavir doses were available, these were considered as hints. However, upon extended exposure to high doses of ritonavir, different types of interaction are likely to take place.

^4^ The severity of interaction may slightly differ between oral and parenteral administration of midazolam with less strong effects expected for parenteral route.

|  | Risk of interactions is arguably very low considering that different pharmacokinetic and pharmacodynamic pathways are involved, although no direct evidence is available |
| --- | --- |
|  | Available data showing very low risk of interactions |
|  | Low risk of interactions |
|  | Moderate risk of interactions |
|  | High risk of interactions, which may include strong alterations in drug exposure or pharmacodynamic synergies. Here we also classify interactions reported in the package insert |

**Narrative synthesis of the literature that guided the discussion**

Note: all the elements reported in this section have been extracted from the studies included after the selection process, plus the additional material that informed the discussion of the working group (see above). All references can be found in the main text.

*Drug-drug interactions*

In patients with COVID-19 the risks associated with drug-drug interactions involving psychotropic medications may be prominent for several reasons. Firstly, COVID-19 has been associated with systemic inflammation processes and elevated inflammation biomarkers, such as elevated C-reactive protein (CRP), that can crucially affect the bioavailability of several psychotropic medications, leading to increased plasma levels and possible toxicity. Specifically, inflammation can affect the activity of cytochrome (CYP) 450 isoenzymes, mainly CYP1A2 and 3A4, that are involved in the metabolism of many psychotropic medications. Second, COVID-19 may be lead to impaired liver functioning, which can, in turn, alter drug disposition. Third, some of the off-label medications used in the COVID-19 treatment, such as antiviral agents, have poorly understood properties on the activity of CYP isoenzymes that can crucially alter the bioavailability of co-medications, including psychotropic medications. On the other hand, psychotropic medications include both strong cytochrome inducers (e.g. carbamazepine) and inhibitors (e.g. paroxetine) that can affect the levels of the COVID-19 medications and, thus, the safety of medical treatments. Fourth, many hospitalised patients with COVID-19 undergo abrupt smoking cessation, and this can rapidly affect the plasma level of medical and psychotropic medications due to the inducing effects of smoking on CYP1A2 activity. Fifth, combinations of psychotropic medications with COVID-19 medications may be associated with pharmacodynamic interactions, such as QTc prolongation, immunity abnormalities and coagulation abnormalities, which will be discussed in the following sections.

*Respiratory risk*

The occurrence of bilateral interstitial pneumonia is considered the key clinical feature of COVID-19 and the major cause of death. This is associated with hypoxic respiratory distress related to impaired regulation of pulmonary blood flow, which can rapidly evolve into a full-blown Acute Respiratory Distress Syndrome (ARDS).

There is debate around the risk for impaired respiratory function associated with psychotropic medications. Although benzodiazepines are generally considered at risk for respiratory suppression by both inhibiting the bulbar respiratory centre and exerting a myorelaxant effect on respiratory muscles, available data are inconclusive. Their effect on respiratory suppression is notably lower as compared with barbiturates or other neuromuscular blocking agents, even in overdose. However, available evidence suggests that there is a relevantly higher risk in patients with acute respiratory distress and in the elderly, possibly because of pre-existing comorbidities, co-treatment with other medications impairing respiration (e.g. opioids), a slower elimination of drugs and the consequent risk of accumulation. The risk of respiratory distress is related to intrinsic sedative proprieties of different agents, longer half-life, and higher prescribed dose, while shorter half-life agents are at lower risk (e.g. etizolam, oxazepam, lorazepam). Data from randomized trials showed no relevant impact on respiratory outcomes in people with COPD treated with benzodiazepines for insomnia, although the pooled sample size was relatively small.

Antipsychotics are associated with an increased risk of respiratory, thoracic and mediastinal serious adverse events according to data from randomized trials. Antipsychotics can increase the risk of respiratory distress, especially in patients with pre-existing respiratory impairment. The risk is higher with highly sedative agents (with strong antihistaminic and anticholinergic profiles, e.g. chlorpromazine), particularly at high doses and when combined together. In case of psychomotor agitation requiring rapid tranquilization with antipsychotics (e.g. hyperkinetic delirium or dementia-related agitation), the risk for acute extrapyramidal symptoms (e.g. dystonia, with possible impaired swallowing and consequent risk of aspiration) and reduced mobility can notably worsen respiratory distress.

Antidepressants, and particularly SSRIs, are generally considered safe in terms of respiratory distress, and are recommended for the treatment of depression in people with physical illnesses, including those with pre-existing respiratory impairment such as chronic obstructive pulmonary disease (COPD).

Although data from randomized trials did not show an increased risk of respiratory distress and overall mortality in patients with COPD (including elderly patients) exposed to SSRIs and TCAs, data from a recent, large observational study showed a higher risk for COPD worsening or COPD-related hospitalization and mortality in older patients taking SSRIs and SNRIs versus those not exposed.

Mood stabilizers have mild-to-moderate sedative profiles, and there is no evidence of a relevant risk for sedation and respiratory distress.

*Cardiovascular risk*

People with COVID-19 are likely to have several cardiovascular risk factors, including: (a) old age; (b) comorbid cardiovascular diseases, including ischemic heart disease, atrial fibrillation and hypertension; (c) use of medical treatments with QTc prolonging properties, often in combination (e.g. antivirals, chloroquine/hydroxychloroquine and antibiotics); (d) possible direct cardiotoxic effects of COVID-19; (e) electrolyte alterations related to abnormal respiratory gas exchange. Both the magnitude of QTc prolongation and characteristics of the population (particularly pre-existing heart disease, female sex, bradycardia, hypokalemia and other electrolyte abnormalities) are the most important determinants of risk for TdP.

Antipsychotics have been shown to be associated with serious cardiovascular events according to data from observational studies assessing sudden cardiac death, myocardial infarction and stroke, while data from randomized trials confirmed an increased risk of QTc prolongation for a number of antipsychotics, but not a higher risk of serious cardiac and vascular adverse events. The network meta-analysis by Huhn at al. 2019 included short-term randomized trials in people with schizophrenia and showed a statistically significant risk of QTc prolongation for sertindole, amisulpride, ziprasidone, iloperidone, asenapine, risperidone, olanzapine, quetiapine as compared to placebo (ordered from higher to lower risk). A safer profile emerged for lurasidone, brexpiprazole, cariprazine, aripiprazole, paliperidone and haloperidol, with no significant differences as compared to placebo. Although QTc prolongation is commonly used in clinical practice to estimate the arrhythmogenic risk, its accuracy in predicting severe arrhythmias, such as Torsade de Pointes (TdP), has been extensively debated. Antipsychotics combination and higher cumulative doses might contribute to QTc prolongation. The differential risk of QTc prolongation of antipsychotics are not entirely consistent across different data sources and study designs. In general, the risk of QTc prolongation should not be neglected for any antipsychotic, including aripiprazole, although its predictive proprieties on TdP is still unclear.

Data from randomized studies in people with ischemic heart disease did not show an increased risk of cardiovascular mortality and nonfatal cardiac events for antidepressants (particularly SSRIs). On the other hand, data from observational studies showed an increased risk of coronary heart disease for tricyclic antidepressants (TCAs), but not SSRIs and antidepressants as a class, while SSRIs but not TCAs were associated with an increased risk for cerebrovascular disease. Tricyclic antidepressants and, to a lesser extent, citalopram, escitalopram and venlafaxine, have been associated with QTc prolongation, with a possibly higher risk in older patients.

The risk of arrhythmias is generally considered to be low for most mood stabilizers and benzodiazepines, also according to the summaries of product characteristics, with the possible exception of lithium, which has been associated with both benign electrocardiographic changes and cases of ventricular arrhythmia and sudden cardiac death.

*Risk of infections*

There is consistent evidence that COVID-19 can target multiple organs through the dysregulation of immunity and inflammation response. Fulminant cytokine storm syndromes resembling secondary haemophagocytic lymphohistiocytosis (sHLH) observed in systemic virosis and bacterial sepsis have been described, and generally the severity of inflammatory parameters (such as IL-6) is associated with fatality risk. On these basis, immunosuppressive therapies have been proposed for both treatment and prevention of complications.

An interplay between psychotropic medications, immunity and inflammation has been largely shown, although there is debate about the clinical implications. Antipsychotics have been associated with decreased pro-inflammatory cytokine levels, possibly exerting a normalizing activity on underlying immune balance dysfunctions, and immunosuppressive proprieties, such as blood dyscrasias and altered production of antibodies (particularly in second-generation antipsychotics). The risk of neutropenia and agranulocytosis is about 1% for clozapine in the general population, and up to 3% in the elderly, while for phenothiazines the risk is about 0.1%, while for other medications sparse reports are available. The risk for clozapine is higher in the first three months of treatment and might increase when co-administered with some antibiotics (i.e. β-lactam antibiotics and sulfamethoxazole-trimethoprim), proton pump inhibitors and other gastrointestinal agents, and carbamazepine, for which a clear contraindication has been issued. Furthermore, both first- and second-generation antipsychotics have been associated with a higher risk of pneumonia in observational studies. Data from randomized trials including mostly second-generation antipsychotics showed a higher risk of infections. This risk might be particularly relevant for clozapine. Apart from several immunity abnormalities, multiple mechanisms may contribute, including reduced clearance of the airways (related to central sedation and inhibition of cough), impaired chest movements and swallowing due to extrapyramidal symptoms, and sialorrea. This risk has been observed for both first- and second-generation antipsychotics, and is particularly relevant for clozapine. Moreover, antipsychotics are associated with the risk Neuroleptic Malignant Syndrome (NMS), a life-threatening condition that is estimated to occur rarely (prevalence of about 0.01%), and characterized by fever, muscle rigidity, and altered mental status. Similarly to other critical medical conditions, patients with COVID-19 might have an increased risk of NMS because of multiple risk factors, such as dehydration, physical exhaustion, electrolyte alterations and delirium.

Antidepressants have been shown to exert a systemic anti-inflammatory effect, which may contribute to their therapeutic effect. However, little is known about their possible positive or negative effects on systemic infections. In vitro studies showed a protective effect against bacteria and fungi, while clinical data are sparse. Generally, antidepressants are considered safe in terms of risk of infections, however there are data supporting a possible association between antidepressants and the infection of Clostridium difficile.

Pharmacovigilance data showed a possible association between fluoxetine and interstitial pneumonia, although the clinical risk, the risk of other SSRIs, and the implications for practice are unclear.

Tricyclic antidepressants (i.e. clomipramine and imipramine), have been associated with possible blood dyscrasias, including neutropenia.

A recent meta-analysis of observational studies showed that benzodiazepines and related drugs were associated with a higher risk of pneumonia, and the association was maintained for both older and younger patients; short-term and long-term use; short-, intermediate- and long-term acting agents; current and recent users (while past users were not at increased risk).

Among mood stabilizers, carbamazepine and oxcarbazepine are at higher risk for neutropenia and have been associated with systemic lupus erythematosus (SLE)-like syndrome (with an arguably low risk). Sodium valproate has been associated with leucopenia, and lamotrigine with potentially serious autoimmune rash. Lithium appears to be free from relevant immunological effects.

*Coagulation risk*

Blood hypercoagulability has been consistently reported in patients with COVID-19, and its severity is associated with higher risk of complications and mortality. The underlying mechanism of vascular inflammation and endothelial dysfunction is ubiquitous, and coagulation profile abnormalities include increased D-Dimer levels, PT and aPTT prolongation, increased fibrin degradation products, and thrombocytopenia. Manifestations of venous thromboembolism can occur at different degrees of severity, up to the life-threatening condition of disseminated intravascular coagulation (DIC). On these basis, low molecular weight heparin has been suggested as an effective prophylaxis since early stages of disease.

Meta-analysis of observational studies showed that antipsychotics are associated with an increased risk of thromboembolism, although it is not clear which medications are at higher risk.

Meta-analysis of observational studies showed that antidepressants are associated with various altered hemostasis parameters, and an increased risk of bleeding at different sites. The risk is higher for tricyclic antidepressants, although the risk of SSRIs can be relevant when other risk factors are in place (e.g. old age, anticoagulant therapy, major surgery).

A recent systematic review and meta-analysis of observational studies showed that both people with depression and the use of antidepressants are associated with an increased risk of venous thromboembolism, and this association was confirmed for TCAs, SSRIs and other antidepressants.

Although systematic reviews on the risk of coagulation abnormalities are not available for mood stabilizers and benzodiazepines, the risk for pro- or anticoagulant effect is likely to be low according to the summaries of product characteristics.

*Risk of delirium*

Although epidemiological data are preliminary, delirium has been described as frequent manifestation of COVID-19, associated with unfavourable prognosis. Old age, medical comorbidities, dementia, and multiple pharmacological treatments are well known risk factors for both delirium and COVID-19 severity. Neurotropic mechanisms of COVID-19 have been also hypothesized. Furthermore, many of the experimental medical treatments use for COVID-19 have a well-known risk for neuropsychiatric side effects (e.g. antimalarial and antiviral medications, interferons, corticosteroids), and may represent an additional risk.

Some psychotropic medications are also known to act as reversible contributing factor for delirium. In particular, benzodiazepines, antidepressants with anticholinergic proprieties (mainly tricyclic antidepressants, but possibly also paroxetine) and lithium are considered at high risk according to data from observational studies. Anticholinergic medications are often a precipitating factor, and are associated with delirium severity. It has been estimated that medications alone might account for up to 40% of cases of delirium. Data from a recent meta-analysis of randomized trials showed that olanzapine and risperidone were effective in preventing delirium as compared to placebo or treatment as usual, while midazolam increased the incidence of delirium.

**Table S9. Evidence to decision framework**

|  | **Judgment of the working group** | **Supporting information** |
| --- | --- | --- |
| Is the problem a priority? | Yes | - People with COVID-19 might frequently experience new onset or exacerbation of psychiatric manifestations in response to the communication of the diagnosis, the need for forced isolation, the presence of severely distressing medical symptoms, and possible risk of death. - Intensive care support and experimental medical treatments with psychiatric side effects (e.g. antimalarials) might be an additional risk factor for the onset psychiatric symptoms and altered states of consciousness. - Psychotropic medications have well-known safety issues, which are possibly worse in people with severe medical condisions who may be exposed to off-label medications with unclear benefits and harms. - Evidence on the safe management of psychotropic medications in people with COVID-19, and, in general, in severely ill patients is lacking. |
| How substantial are the desirable anticipated effects? | Varies | - As this document is focused on safety issues only, the beneficial effects of psychotropic medications were not considered. - The working group agrees on the fact that desirable effects should be carefully weighed on a case-by-case basis, after a detailed assessment of psychiatric symptoms. - The risk of inappropriate prescription of psychotropic medications is common, and should be taken into due account. |
| How substantial are the undesirable anticipated effects? | Moderate-to-large | - See “Narrative synthesis of the evidence” - In general, all classes of psychotropic medications may have clinically relevant safety issues in people with COVID-19, due to the COVID-related vulnerability and the potential interaction with off-label medical treatments used for COVID-19. - The severity of safety issues relevantly changes according to demographic factors (e.g. age), severity of the coronavirus infection, and co-occurring somatic vulnerabilities (e.g. pre-existing cardiovascular or resiratory conditions). |
| What is the overall certainty of the evidence of effects? | Low-to-moderate | - The AMSTAR-2 tool showed relevant heterogeneity in the methodological quality of the included systematic reviews. - Some of the included systematic reviews provided other measures of the certainty/credibility of data, which were narratively appraised in the working group discussions. - A formal assessment of the certaint with the GRADE approach was not performed, in line with the WHO Rapid Advice Guidelines for public health emergencies. |
| Is there important uncertainty about or variability in how much people value the main outcomes? | No important uncertainty or variability | - No formal analysis was performed on this topic, and no key stakeholders were included in the working group. - According to the working group clinical expertise, there was broad agreement on the fact that safety issues related to psychotropic medications are considered of utmost value and importance for patients, family members, caregivers, infective disease specialists and other medical doctors dealing with the COVID-19 pandemic. |
| Does the balance between desirable and undesirable effects favor the intervention or the comparison? | Varies | - Assessing the balance between desirable and undesirable effects might be particularly challenging in this severely ill population. The decision of withdrawing, switching, or adjusting the dose of a psychotropic medication should be carefully weighed on a case-by-case basis, after a thorough assessment of psychiatric symptoms, severity of COVID-19, and the possibile beneficial effect of off-label medical treatments for COVID-19. |
| How large are the resource requirements (costs)? | Moderate savings | - No formal cost-effectiveness analyses were performed. - Acquisition costs are generally very low for most psychotropic medications. - Moderate savings due to a reduction of medical complications might be expected. |
| What would be the impact on health equity? | Probably increased | - Recommendations formulated by the working group will be an easily accessible tool for any type of doctors at a global level, includung those working in low- and middle-income settings, and this represents a facilitator for the applicability of the recommendations in clinical practice. |
| Is the intervention acceptable to key stakeholders? | Probably yes | - According to the working group clinical expertise, an appropriate assessment and management of the safety issues related to psychotropic medications in people with COVID-19 does not imply problems of acceptability for patients, family members, other caregivers. - Appropriate psychosocial interventions should always be considered as a valuable tool to reduce the emotional distress in medically ill patients and in their family members and caregivers. This principle is especially important when treatment with psychotropic medications is not feasible. |
| Is the intervention feasible to implement? | Probably yes | - No relevant barriers to implementation of these practical recommendation emerged in terms of costs, acceptability and values to key stakeholders. - Managing psychotropic medications by adjusting the dose and providing monitoring is probably feasible in most cases. - Withdrawing or switching a psychotropic medications might require consultation with a specialist, particularly in case of long-standing maintenance treatments (e.g. lithium) and for medications with high potenital of withdrawal effects (e.g. benzodiazepines and some antidepressants, i.e. venlafaxine). - Limited number and duration of face-to-face visits due to the isolation for the risk of infection might lower the feasibility of psychiatric clinical assessment of people with COVID-19. |

**Recommendations**

1. The risk and severity of drug-drug pharmacokinetic and pharmacodynamic interactions between COVID-19 medical treatments and psychotropic medications should always be assessed, taking into account the additional vulnerability related to the underlying medical condition (e.g. cardiovascular conditions increasing the risk of QTc prolongation).
2. In case of high-risk interactions, the combination should be avoided if possible. In case of moderate-risk interactions, dose adjustments, psychotropic medication withdrawal, or switch to a safer medication, should be considered. In case of low-risk interactions, regular monitoring should be provided, with dose adjustments as clinically appropriate. In case of very low-risk interaction, regular monitoring is suggested (see Table 2 and Supplement).
3. An estimation of psychotropic-related risk of respiratory depression should systematically take into account: (a) the intrinsic sedative proprieties of psychotropic medications, their half-life (higher risk for longer half-life), the dose, and the occurrence of other aspects possibly impairing respiration (e.g. reduced motility, sialorrhea); (b) pharmacokinetic interactions raising plasma levels of sedative medications (e.g. lopinavir/ritonavir combined with quetiapine), and pharmacodynamic interactions (e.g. co-treatments with opioids); (c) pre-existing respiratory impairment (e.g. COPD) and degree of COVID-19-related respiratory depression.
4. Antipsychotic medications are at risk of worsening respiratory function in people with COVID-19, particularly at high doses and when used in combination. Antipsychotics with highly sedative profiles should be avoided or used short-term.
5. The risk of respiratory impairment associated with benzodiazepines in the general population is debated, but might be particularly relevant in elderly patients with COVID-19 and pre-existing comorbidities (e.g. COPD). Benzodiazepines should be avoided or used short-term (e.g. control of acute agitation), preferring those with shorter half-life (e.g. etizolam, oxazepam, lorazepam). Although antidepressants are generally considered safe in terms of respiratory impairment, caution is advised as data are controversial.
6. An estimation of psychotropic-related risk of cardiovascular events should systematically take into account: (a) the intrinsic QTc-prolonging proprieties of psychotropic medications, their cumulative dose and use in combination; (b) pharmacokinetic interactions possibly raising plasma levels of QTc-prolonging medications and pharmacodynamic interactions (e.g. co-treatments with antivirals, chloroquine, hydroxychloroquine, opioids); (c) pre-existing cardiovascular conditions (in particular, ischemic heart disease) and COVID-19-related cardiovascular conditions.
7. For interactions with low-to-moderate risk of QTc prolongation, an adjustment towards a lower dose of one or both medications is generally required, along with regular electrocardiogram monitoring. In case these interactions add up with other risk factors for QTc prolongation (e.g. cardiovascular comorbidities, electrolyte abnormalities) medications at risk should be avoided, or withdrawn, or switched to safer medications, as clinically appropriate.
8. Antipsychotics, benzodiazepines and some mood stabilizers may be associated with an increased risk of secondary infections in people with COVID-19, and possibly with an unfavorable course of systemic infections. The risk is likely to be particularly relevant for clozapine, carbamazepine and oxcarbazepine. Regular monitoring is therefore indicated.
9. In people with COVID-19, both antipsychotics and antidepressants might increase the risk of thromboembolism, particularly in the elderly. In people with COVID-19 taking heparin prophylaxis, antidepressants might increase the risk of bleeding, with a higher risk for serotoninergic agents (i.e. SSRIs and SNRIs), especially in elderly patients. Regular monitoring is indicated. In case there are additional risk factors for bleeding (e.g. other coagulation abnormalities, old age, anticoagulant therapy, major surgery), a dose adjustment or withdrawing the medications at risk should considered, as clinically appropriate.
10. In people with COVID-19 and known risk factors for delirium (e.g. old age, dementia, multiple comorbidities), the use of agents with anticholinergic properties (e.g. tricyclic antidepressants and paroxetine), benzodiazepines (particularly midazolam), and lithium should generally be avoided.
11. In patients with COVID-19 who are already in treatment with psychotropic medications, an accurate assessment of current psychiatric symptoms and past psychiatric history is important in order to review the need of continuing treatment and its dose.
12. In addition to psychotropic medications, and when drug treatment is clinically inappropriate, clinicians should carefully assess whether adequate supportive psychosocial interventions are provided, including electronically-delivered interventions.

**Table S10. AGREE Reporting Checklist**


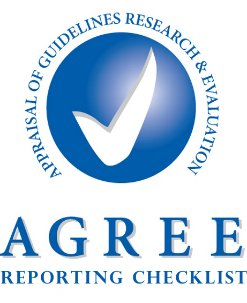


**AGREE Reporting Checklist**

2016

*This checklist is intended to guide the reporting of clinical practice guidelines.*

| **CHECKLIST ITEM AND DESCRIPTION** | **REPORTING CRITERIA** | **Page #** |
| --- | --- | --- |
| ***DOMAIN 1: SCOPE AND PURPOSE*** | | |
| **1. OBJECTIVES**  *Report the overall objective(s) of the guideline. The expected health benefits from the guideline are to be specific to the clinical problem or health topic.* | Health intent(s) (i.e., prevention, screening, diagnosis, treatment, etc.)  Expected benefit(s) or outcome(s)  Target(s) (e.g., patient population, society) | Background (p.4-5) |
| **2. QUESTIONS**  *Report the health question(s) covered by the guideline, particularly for the key recommendations.* | Target population  Intervention(s) or exposure(s)  Comparisons (if appropriate)  Outcome(s)  Health care setting or context | Methods (p. 5-6) and Supplement (PICO question and framework) |
| **3. POPULATION**  *Describe the population (i.e., patients, public, etc.) to whom the guideline is meant to apply.* | Target population, sex and age  Clinical condition (if relevant)  Severity/stage of disease (if relevant)  Comorbidities (if relevant)  Excluded populations (if relevant) | Methods (p. 5-6) and Additional file (PICO question and framework) |
| ***DOMAIN 2: STAKEHOLDER INVOLVEMENT*** | | |
| **4. GROUP MEMBERSHIP**  *Report all individuals who were involved in the development process. This may include members of the steering group, the research team involved in selecting and reviewing/rating the evidence and individuals involved in formulating the final recommendations.* | Name of participant  Discipline/content expertise (e.g., neurosurgeon, methodologist)  Institution (e.g., St. Peter’s hospital)  Geographical location (e.g., Seattle, WA)  A description of the member’s role in the guideline development group | Methods (p. 5-6) and Additional file (Working Group composition) |
| **5. TARGET POPULATION PREFERENCES AND VIEWS**  *Report how the views and preferences of the target population were sought/considered and what the resulting outcomes were.* | Statement of type of strategy used to capture patients’/publics’ views and preferences (e.g., participation in the guideline development group, literature review of values and preferences)  Methods by which preferences and views were sought (e.g., evidence from literature, surveys, focus groups)  Outcomes/information gathered on patient/public information  How the information gathered was used to inform the guideline development process and/or formation of the recommendations | Additional file (Evidence-to-Decision framework) |
| **6. TARGET USERS**  *Report the target (or intended) users of the guideline.* | The intended guideline audience (e.g. specialists, family physicians, patients, clinical or institutional leaders/administrators)  How the guideline may be used by its target audience (e.g., to inform clinical decisions, to inform policy, to inform standards of care) | Background (p.4-5) |
| ***DOMAIN 3: RIGOUR OF DEVELOPMENT*** | | |
| **7. SEARCH METHODS**  *Report details of the strategy used to search for evidence.* | Named electronic database(s) or evidence source(s) where the search was performed (e.g., MEDLINE, EMBASE, PsychINFO, CINAHL)  Time periods searched (e.g., January 1, 2004 to March 31, 2008)  Search terms used (e.g., text words, indexing terms, subheadings)  Full search strategy included (e.g., possibly located in appendix) | Methods (p. 6-8) and Additional file (Search strategy; List of included and excluded studies) |
| **8. EVIDENCE SELECTION CRITERIA**  *Report the criteria used to select (i.e., include and exclude) the evidence. Provide rationale, where appropriate.* | Target population (patient, public, etc.) characteristics  Study design  Comparisons (if relevant)  Outcomes  Language (if relevant)  Context (if relevant) | Methods (p. 6-8) and Additional file (PICO question and framework; Search strategy; List of included and excluded studies) |
| **9****. STRENGTHS & LIMITATIONS OF THE EVIDENCE**  *Describe the strengths and limitations of the evidence. Consider from the perspective of the individual studies and the body of evidence aggregated across all the studies. Tools exist that can facilitate the reporting of this concept.* | Study design(s) included in body of evidence  Study methodology limitations (sampling, blinding, allocation concealment, analytical methods)  Appropriateness/relevance of primary and secondary outcomes considered  Consistency of results across studies  Direction of results across studies  Magnitude of benefit versus magnitude of harm  Applicability to practice context | Results (p. 7-13); Table 1; Additional file (Narrative synthesis of the evidence; Evidence-to-Decision framework) |
| **10. FORMULATION OF RECOMMENDATIONS**  *Describe the methods used to formulate the recommendations and how final decisions were reached. Specify any areas of disagreement and the methods used to resolve them.* | Recommendation development process (e.g., steps used in modified Delphi technique, voting procedures that were considered)  Outcomes of the recommendation development process (e.g., extent to which consensus was reached using modified Delphi technique, outcome of voting procedures)  How the process influenced the recommendations (e.g., results of Delphi technique influence final recommendation, alignment with recommendations and the final vote) | Methods (p. 7) and Additional file (Evidence-to-Decision framework) |
| **11. CONSIDERATION OF BENEFITS AND HARMS**  *Report the health benefits, side effects, and risks that were considered when formulating the recommendations.* | Supporting data and report of benefits  Supporting data and report of harms/side effects/risks  Reporting of the balance/trade-off between benefits and harms/side effects/risks  Recommendations reflect considerations of both benefits and harms/side effects/risks | Results (p. 7-13); Table 1 and 2; Additional file (Narrative synthesis of the evidence; Evidence-to-Decision framework) |
| **12. LINK BETWEEN RECOMMENDATIONS AND EVIDENCE**  *Describe the explicit link between the recommendations and the evidence on which they are based.* | How the guideline development group linked and used the evidence to inform recommendations  Link between each recommendation and key evidence (text description and/or reference list)  Link between recommendations and evidence summaries and/or evidence tables in the results section of the guideline | Discussion (p. 16-18) and Additional file (Evidence-to-Decision framework) |
| **13. EXTERNAL REVIEW**  *Report the methodology used to conduct the external review.* | Purpose and intent of the external review (e.g., to improve quality, gather feedback on draft recommendations, assess applicability and feasibility, disseminate evidence)  Methods taken to undertake the external review (e.g., rating scale, open-ended questions)  Description of the external reviewers (e.g., number, type of reviewers, affiliations)  Outcomes/information gathered from the external review (e.g., summary of key findings)  How the information gathered was used to inform the guideline development process and/or formation of the recommendations (e.g., guideline panel considered results of review in forming final recommendations) | Discussion (p. 18-19) |
| **14. UPDATING PROCEDURE**  *Describe the procedure for updating the guideline.* | A statement that the guideline will be updated  Explicit time interval or explicit criteria to guide decisions about when an update will occur  Methodology for the updating procedure | Discussion (p. 19) |
| ***DOMAIN 4: CLARITY OF PRESENTATION*** | | |
| **1****5. SPECIFIC AND UNAMBIGUOUS RECOMMENDATIONS**  *Describe which options are appropriate in which situations and in which population groups, as informed by the body of evidence.* | A statement of the recommended action  Intent or purpose of the recommended action (e.g., to improve quality of life, to decrease side effects)  Relevant population (e.g., patients, public)  Caveats or qualifying statements, if relevant (e.g., patients or conditions for whom the recommendations would not apply)  If there is uncertainty about the best care option(s), the uncertainty should be stated in the guideline | Results (p. 14-16) |
| **16. MANAGEMENT OPTIONS**  *Describe the different options for managing the condition or health issue.* | Description of management options  Population or clinical situation most appropriate to each option | Results (p. 14-16) |
| **17. IDENTIFIABLE KEY RECOMMENDATIONS**  *Present the key recommendations so that they are easy to identify.* | Recommendations in a summarized box, typed in bold, underlined, or presented as flow charts or algorithms  Specific recommendations grouped together in one section | Results (p. 14-16) |
| ***DOMAIN 5: APPLICABILITY*** | | |
| **1****8. FACILITATORS AND BARRIERS TO APPLICATION**  *Describe the facilitators and barriers to the guideline’s application.* | Types of facilitators and barriers that were considered  Methods by which information regarding the facilitators and barriers to implementing recommendations were sought (e.g., feedback from key stakeholders, pilot testing of guidelines before widespread implementation)  Information/description of the types of facilitators and barriers that emerged from the inquiry (e.g., practitioners have the skills to deliver the recommended care, sufficient equipment is not available to ensure all eligible members of the population receive mammography)  How the information influenced the guideline development process and/or formation of the recommendations | Additional file (Evidence-to-Decision framework) |
| **19. IMPLEMENTATION ADVICE/TOOLS**  *Provide advice and/or tools on how the recommendations can be applied in practice.* | Additional materials to support the implementation of the guideline in practice.  For example:   - Guideline summary documents - Links to check lists, algorithms - Links to how-to manuals - Solutions linked to barrier analysis (see Item 18) - Tools to capitalize on guideline facilitators (see Item 18) - Outcome of pilot test and lessons learned | Additional file; Table 2 and extended drug-drug interaction table |
| **20. RESOURCE IMPLICATIONS**  *Describe any potential resource implications of applying the recommendations.* | Types of cost information that were considered (e.g., economic evaluations, drug acquisition costs)  Methods by which the cost information was sought (e.g., a health economist was part of the guideline development panel, use of health technology assessments for specific drugs, etc.)  Information/description of the cost information that emerged from the inquiry (e.g., specific drug acquisition costs per treatment course)  How the information gathered was used to inform the guideline development process and/or formation of the recommendations | Additional file (Evidence-to-Decision framework) |
| **21. MONITORING/ AUDITING CRITERIA**  *Provide monitoring and/or auditing criteria to measure the application of guideline recommendations.* | Criteria to assess guideline implementation or adherence to recommendations  Criteria for assessing impact of implementing the recommendations  Advice on the frequency and interval of measurement  Operational definitions of how the criteria should be measured | Discussion (p. 18-19) |
| ***DOMAIN 6: EDITORIAL INDEPENDENCE*** | | |
| **2****2. FUNDING BODY**  *Report the funding body’s influence on the content of the guideline.* | The name of the funding body or source of funding (or explicit statement of no funding)  A statement that the funding body did not influence the content of the guideline | Declarations (p. 20-23) |
| **23. COMPETING INTERESTS**  *Provide an explicit statement that all group members have declared whether they have any competing interests.* | Types of competing interests considered  Methods by which potential competing interests were sought  A description of the competing interests  How the competing interests influenced the guideline process and development of recommendations | Declarations (p. 20-23) |

From:

Brouwers MC, Kerkvliet K, Spithoff K, on behalf of the AGREE Next Steps Consortium. The AGREE Reporting Checklist: a tool to improve reporting of clinical practice guidelines. *BMJ* 2016;352:i1152. doi: 10.1136/bmj.i1152.

For more information about the AGREE Reporting Checklist, please visit the AGREE Enterprise website at <http://www.agreetrust.org>.
